# Hemiarthroplasty vs. proximal femoral nail fixation in unstable pertrochanteric fractures: an updated systematic review and meta-analysis

**DOI:** 10.3389/fsurg.2026.1782908

**Published:** 2026-03-02

**Authors:** Ming-Ming Zhang, Shuang-Shuang Yuan, Hong-Hua Dong, Chao Wang

**Affiliations:** Department of Orthopedics, The Yancheng School of Clinical Medicine of Nanjing Medical University, Yancheng Third People's Hospital, Yancheng, Jiangsu, China

**Keywords:** hemiarthroplasty, hip fracture (HFr), hip surgery, pertrochanteric fractures, proximal femoral nail

## Abstract

**Background:**

Pertrochanteric fractures are common and challenging for surgeons, especially in unstable conditions. Proximal femoral nails or nails anti-rotation variants are well-established surgical instruments to treat this, but some reports suggest that in unstable situations, hemiarthroplasty provides superior benefits to patients. This study aimed to compare hemiarthroplasty with proximal femoral nails, highlighting the differences to assist surgical decision-making.

**Methods:**

Online databases were searched for eligible studies in accordance with PRISMA guidelines. Results were analyzed across 18 domains, categorized into three branches: function, complication, and perioperative condition and mortality. Effect sizes were calculated, and the heterogeneities thereof were analyzed. We also tested sensitivity, publication biases, and graded certainty of evidence. Finally, comprehensive results were interpreted.

**Results:**

Twenty-seven studies, with a total of 2,517 patients, were included. The hemiarthroplasty performed better than nails in early Harris hip scores, full weight-bearing time, and complication rate related to implants. Proximal femoral nails performed better in terms of surgery duration and superficial infection. No significant differences were observed in final Harris hip scores, general and implant-unrelated complications, mortality, hospital stay, re-operation incidence, bedsores, and deep venous thrombosis. Ambulation time, blood loss, and transfusion incidence showed potential publication bias.

**Conclusion:**

Hemiarthroplasty and proximal femoral nails/nails anti-rotation are effective methods for treating unstable pertrochanteric fractures. No clinically important differences—such as re-operation rates due to implant-related complications—were identified between these two tactics. Surgeons should prioritize internal fixations, barring conditions wherein hemiarthroplasty is essential.

## Introduction

Pertrochanteric fractures are common in patients with osteoporosis due to aging or medical diseases, or in patients encountering high-energy hip trauma. Their increasing prevalence—either in senile women (1, 2) or youths with certain medical or social conditions—underscores the growing economic burden on both the patients and society (3). Over the years, surgical methods and instruments have been continuously invented and modified, with emphasis on patient quality of life and prognosis. Extramedullary internal fixation, after decades of comparison, is now deemed less beneficial than intramedullary internal fixation (4). Among intramedullary options and instrument choices (e.g., gamma nail, proximal femoral nail (PFN), proximal femoral nail anti-rotation (PFNA), InterTAN), PFN and PFNA are the most widely applied. The debate between PFN and PFNA is trivial (5–7) compared to that between extra- and intramedullary fixations, as both instruments yield satisfactory results for patients with pertrochanteric fractures. Nevertheless, in unstable pertrochanteric fractures, surgeons face uncertainty and prepare themselves for revision if internal fixation fails. Hemiarthroplasty (HA) is the conventional salvage surgery following proximal hip instrument failure. However, the fixation–failure–revision procedure compromises prognosis, quality of life, and survival. Surgeons are increasingly presenting the idea of primary rather than revision arthroplasty in unstable pertrochanteric fractures, considering it no longer an extreme, extensive, or aggressive procedure (8–10). Comparisons between HA and PFN/PFNA have persisted for years, with varying conclusions: some favor HA (11, 12) and some favor PFN/PFNA (13, 14), while others report inconclusive (15, 16) or statistically insignificant results (17, 18). Considering the potential selection bias, inappropriate data merging, and heterogeneity explanations, we think it is necessary and important to conduct a comprehensive meta-analysis with all previously eligible studies to evince a convincing relationship between HA and PFN/PFNA.

## Materials and methods

Following the PICOS framework, this study aimed to determine whether hemiarthroplasty (Intervention) leads to better outcomes compared to internal fixation (Comparison) for patients with unstable pertrochanteric fracture (Population). Outcomes were assessed across 18 different domains covering function, complications, and perioperative matters. We included evidence from randomized controlled trials (RCTs), retrospective studies, and prospective cohort studies (study design). We surveyed five central online databases—PubMed, Europe PMC, Web of Science, Embase, and CENTRAL (Cochrane Central Register of Controlled Trials)—for eligible studies. The analysis was conducted according to the PRISMA principles (19). The certainty of evidence was evaluated as per the GRADE system (20). Study quality was assessed with the Cochrane Risk of Bias (RoB) tool (21) and the Newcastle–Ottawa Scale (NOS) (22) for several research designs. To ensure methodological rigor and minimize potential bias, the study selection and data extraction processes were conducted in strict accordance with PRISMA guidelines. Two reviewers independently screened all titles and abstracts, followed by a double-blind full-text review. Data extraction was performed by two independent investigators using a pre-piloted, standardized form. Discrepancies were resolved through consensus or adjudication by a third senior investigator. The five databases were selected to ensure the most comprehensive coverage of mainstream biomedical literature. To uphold the global generalizability of our findings, no language restrictions were applied during the search or screening phases. Furthermore, to mitigate the risk of incorporating low-quality evidence, we strictly restricted our inclusion to peer-reviewed, published literature. While gray literature was acknowledged, its exclusion was a deliberate methodological choice to safeguard the internal validity of the meta-analysis, ensuring that all synthesized data had been subjected to rigorous, independent scholarly scrutiny. This approach minimizes the potential for ascertainment bias and strengthens the overall quality of the evidence base.

### Search strategy

Detailed search strategies are provided in Supplementary Material S1. References cited in reviews were screened and included if eligible.

### Criteria

The inclusion criteria were as follows: (1) English-language literature, or studies with convertible transcripts; (2) patients with unstable pertrochanteric fractures without any collateral damage that could affect surgical tactics (Note: Pertrochanteric fractures were classified according to the AO definition. Many authors or surgeons incorrectly reported these fractures as “intertrochanteric” fractures, possibly for interpretation or translation reasons; therefore, we added the term “intertrochanteric” into the search strategy.); (3) studies comparing HA and PFN/PFNA, with pictures, brand names, or instrument model illustrations; and (4) comparative study designs, e.g., RCT, retrospective studies, or prospective cohort studies, with results that can demonstrate the effect sizes, which are appropriate for synthesis.

The exclusion criteria were are follows: (1) studies unavailable for analysis, letters, reviews, case reports, or ones presenting unconvincing results; (2) pertrochanteric fractures with comorbidities that have a substantial impact on decision-making; (3) studies reporting rare, isolated effect sizes, or insufficient data for research; and (4) studies conflating PFN/PFNA with other internal fixation instruments, HA with total hip arthroplasty, or reporting collective results of mixed instruments or non-specific arthroplasty.

### Data extraction

After screening, data were extracted as follows: (1) author information, publication year, and regions; (2) patient demographics; and (3) results that can represent effect sizes. Here, we extracted 18 results for analysis: 1: Harris hip score (HHS) (initial); 2: Harris hip score (6-month); 3: Harris hip score (final); 4: operative time; 5: superficial infection; 6: re-operation; 7: mortality (final); 8: blood loss; 9: ambulation time; 10: full weight-bearing time; 11: hospital stay; 12: mortality (early); 13: bedsores; 14: deep venous thrombosis (DVT); 15: general complication; 16: implant-related complications; 17: implant-unrelated complications; and 18: blood transfusion. Although some studies presented worthwhile results—such as pedobarographic gait analysis (23) or health-related quality-of-life scores (24)—the rarity of these studies forfeited the possibility of including them unless sufficient sample sizes were guaranteed.

### Quality assessment

RCTs were assessed using the Cochrane RoB tool, with results presented in risk-of-bias graphs and summaries. Retrospective or observational studies were assessed using NOS, with results presented in tables.

Given the inherent complexities and potential for performance bias in surgical interventions—particularly the distinctions between minimally invasive internal fixation and total joint arthroplasty—a conservative evidentiary approach was adopted. We conducted a granular assessment of the included trials, evaluating regional economic status, institutional tier, and the academic capacity of the participating orthopedic departments. To ensure the highest level of stringency, all included studies, including putative RCTs, were appraised as non-randomized studies (NRS). According to the Cochrane-GRADE framework, these studies were initially categorized as “low” certainty. This classification reflects our rigorous methodological thresholds rather than deficiencies in primary study quality. To further investigate potential confounding and treatment indication bias, effect sizes were partitioned into 18 distinct domains for comprehensive subgroup analyses. Furthermore, meta-regression was executed to evaluate extrinsic covariates. The stability of the results across these 18 strata supports the robustness of the pooled estimates and minimizes the risk of biased causal inference.

### Certainty of evidence

We assessed the certainty of evidence of each study included using the GRADE system and ranked it into four levels: very low, low, moderate, and high. Two independent reviewers performed the evaluations, and any disagreements were resolved through consultation with a third reviewer.

### Statistical analysis

We conducted all analyses using the RevMan computer program (version 5.4, The Cochrane Collaboration, 2020) and Comprehensive Meta-analysis (CMA, version 4) software. For continuous variables, we used the inverse variance method for data merging. For dichotomous variables, we used the Mantel–Haenszel method. All studies were analyzed under the random-effects model, and heterogeneity was assessed using the *I*^2^ and prediction intervals (PIs) to draw a final decision.

It should be noted that to rigorously evaluate the variance of true effects, we incorporated PIs for all 18 analyzed domains. While *I*^2^ is a relative ratio representing the proportion of total variation due to heterogeneity, the PI quantifies the absolute range in which the effect of a future study is expected to fall, providing a more robust contextualization of clinical variability. This advanced statistical approach, currently utilized in fewer than 10% of meta-analyses, was applied universally—even across domains with near-zero *I*^2^—to ensure maximum transparency. All pooled estimates were synthesized using a random-effects model, acknowledging that true effects vary across different surgical contexts.

## Results

### Study characteristics

**Figure 1:**
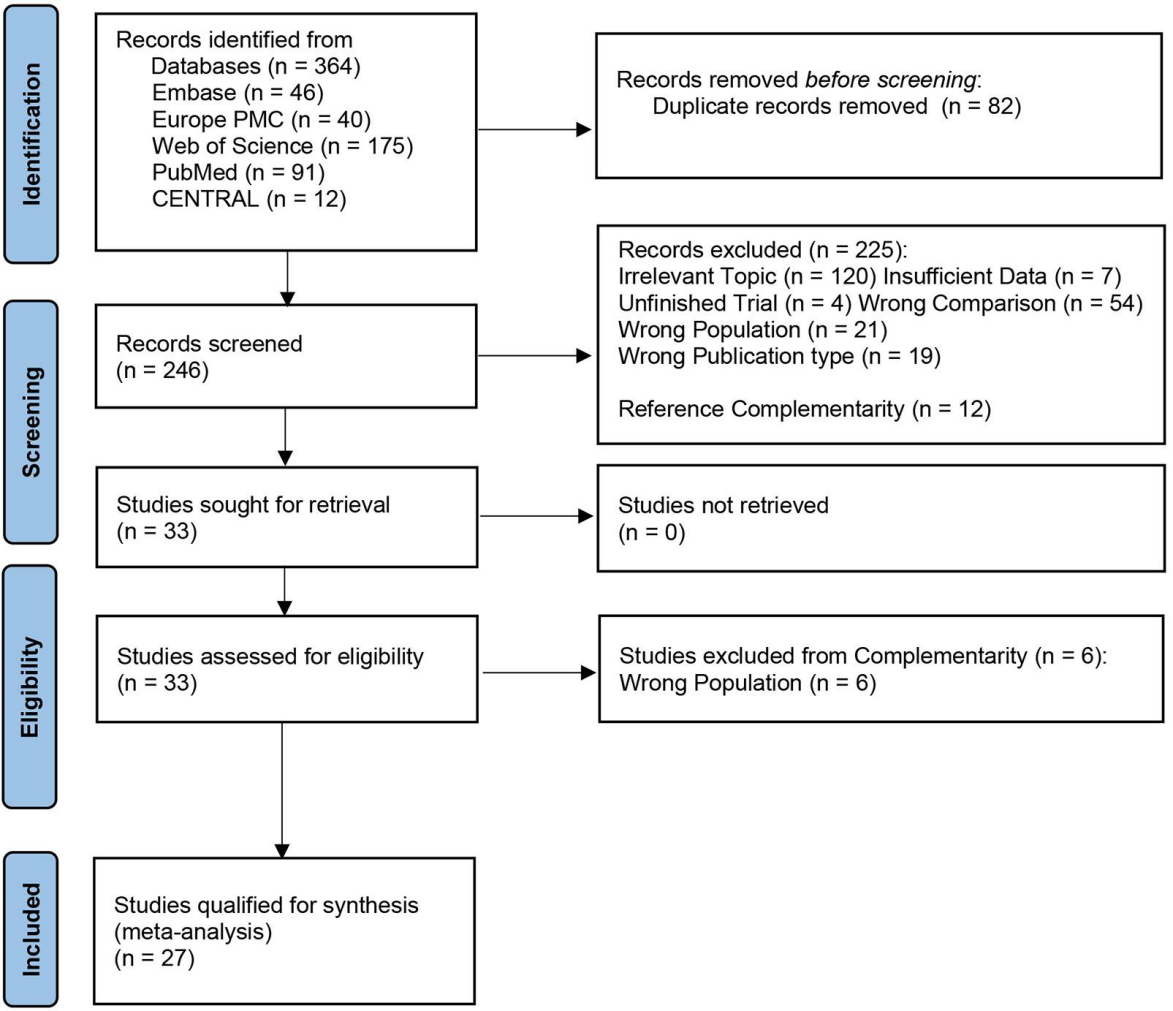
PRISMA flowchart.

**Figure 2:**
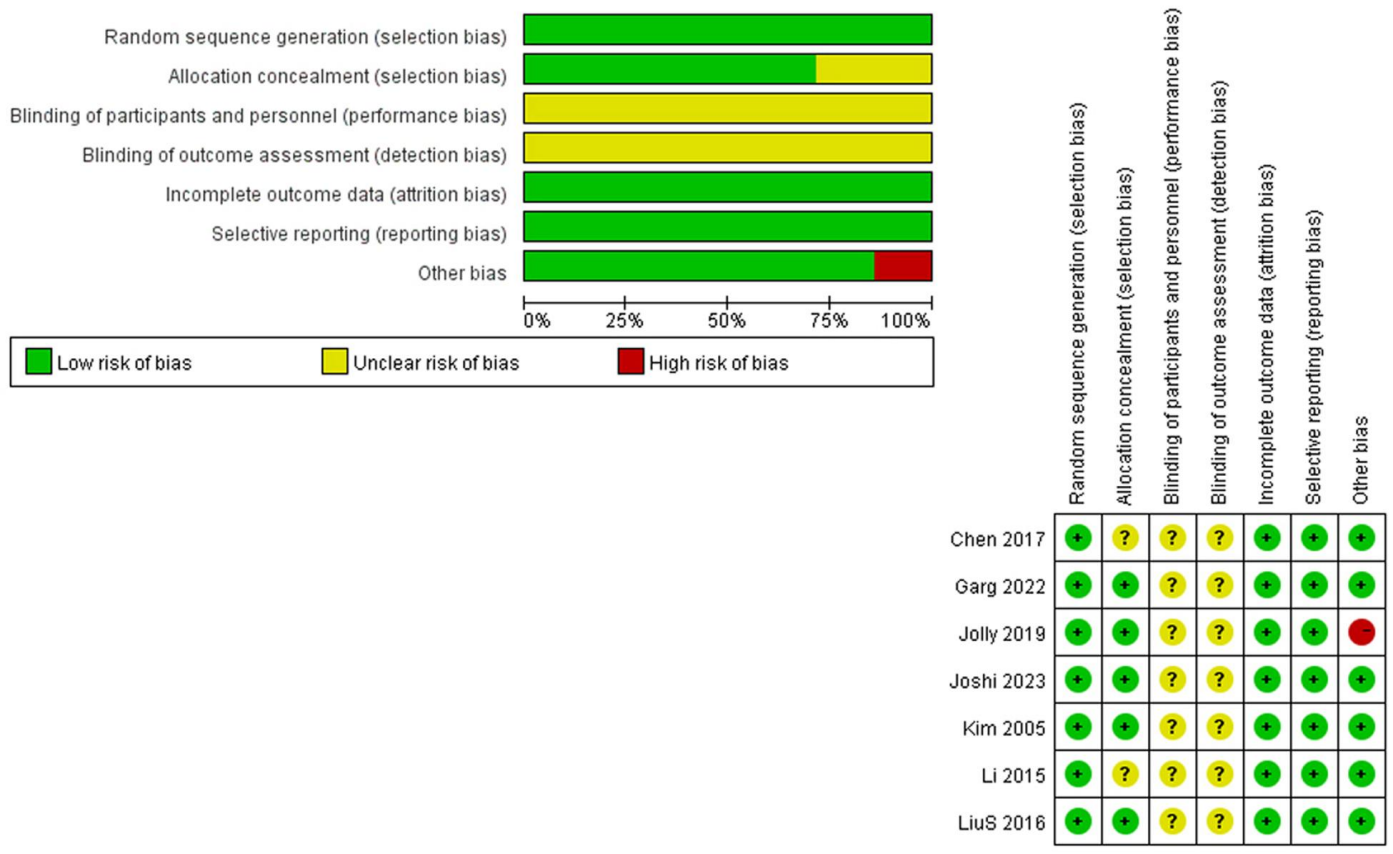
Risk of bias of RCTs.

A total of 364 studies were retrieved from the databases, and 82 duplicates were removed after initial screening. We checked the reviews and included twelve additional studies, leaving 246 studies for full screening. Of these, 229 studies were excluded due to the unavailability of data merging, inadequate sample size or follow-up duration, vague fracture classification or instrument definition, or any reason making studies ineligible. Finally, 29 studies remained; however, two studies reported total hip arthroplasty either mixed with or instead of hemiarthroplasty (25, 26), which were excluded. At last, we selected 27 studies (27–53) for this systematic review and meta-analysis, with details provided in Table 1. The PRISMA flowchart in Figure 1 illustrates the process. Among these 27 studies, seven studies (30, 35, 38, 40, 41, 43, 44) were RCTs, and the remainder were non-RCTs. We defined the unstable fracture as per the traditional Evans–Jensen classification or the AO classification. Both cemented and uncemented hemiarthroplasties were deemed certified surgical treatments. We also listed effect sizes with specific numbers. We verified the quality of RCTs using the RoB method, with risk-of-bias graph and summary presented in Figure 2. The quality of non-RCTs was evaluated using NOS, with results presented in Table 2.

**Table 1:** Characteristics of included studies.

Study	Classification	Surgical tactics I/C	Study design	Outcomes
Agar et al. (2021) ( 31 )	31A2/31A3	UCHA/PFN	Retrospective	3, 4, 6, 7, 8, 11, 17, 18
Cai et al. (2022) ( 32 )	31A2	UCH/PFNA	Retrospective	1, 2, 3, 4, 5, 6, 8, 9, 11, 14, 15, 16, 17, 18
Canbeyli et al. (2021) ( 33 )	31A2.2	CHA/PFNA	Retrospective	5, 7, 12, 16
Çelen and Gazi (2022) (34)	31A2.2/31A2.3	UCHA/PFN	Retrospective	3, 4, 7, 11, 16, 18
Chen et al. (2017) ( 35 )	31A2.2/31A2.3/31A3.1	CHA/PFNA	RCT	3, 4, 8, 9, 11, 15
Çiloğlu et al. (2022) ( 36 )	31A2.2/31A2.3/31A3.3	UCHA/PFNA	Retrospective	4, 5, 6, 7, 10, 12, 14, 15, 16, 17, 18
Huai-dong (2016) (27)	Evans–Jensen III–V	CHA/PFNA	Retrospective	1, 2, 3, 4, 7, 8, 9, 11, 14, 16, 17
Feng et al. (2017) ( 37 )	Evans–Jensen III and IV	CHA/PFNA	Retrospective	1, 3, 5, 10, 12, 13, 15
Garg et al. (2022) ( 38 )	31A2.2/31A2.3	UCHA CHA/PFN	RCT	3, 4, 5, 8, 9, 10, 11, 13
Hussain et al. (2017) ( 39 )	31A2.2/31A2.3/31A3.1	CHA/PFNA	Retrospective	1, 2, 5, 14, 15, 16, 17, 18
Jolly et al. (2019) ( 40 )	Evans–Jensen unstable	CHA/PFN	RCT	1, 2, 3, 4, 5, 7, 8, 10, 12, 13, 14, 16, 17
Joshi et al. (2023) ( 41 )	31A2.2 31A2.3	CHA/PFN	RCT	1, 2, 4, 5, 7, 8, 11, 16
Kilinc and Pazarci (2021) (42)	31A2.2–31A3.3	UCHA/PFNA	Retrospective	4, 5, 6, 7, 8, 16
Kim et al. (2005) ( 43 )	31A2	UCHA/PFN	RCT	3, 4, 5, 6, 7, 8, 12, 14, 16, 17, 18
Li e et al. (2013) ( 46 )	Evans–Jensen unstable	CHA/PFNA	Retrospective	4, 8, 9, 15
Lei (2015) (44)	Evans–Jensen III and IV	CHA/PFNA	RCT	1, 2, 3, 7, 9, 14
Li et al. (2020) ( 45 )	Evans–Jensen III and IV	CHA/PFNA	Retrospective	1, 2, 3, 4, 5, 8, 9, 10, 11, 13, 14, 15, 16, 17
Ming (2012) (29)	Evans–Jensen III–V	CHA/PFNA	Retrospective	4, 8, 9, 15
Chao-Jian (2016) (28)	Evans–Jensen III and IV	CHA/PFNA	RCT	4, 5, 8, 9, 14, 15, 16, 17
Liu et al. (2021) ( 47 )	Evans–Jensen III–V	UCHA/PFNA	Retrospective	1, 4, 8, 9, 14, 15, 16, 17
Chao-Jian 2016 (28)	AO unstable	CHA/PFNA	Retrospective	1, 2, 3, 4, 8, 11, 12, 15, 16, 17, 18
Pang et al. (2013) ( 48 )	Evans–Jensen III–V	CHA/PFNA	Retrospective	4, 8, 10, 15, 16, 17
Song et al. (2022) ( 49 )	Evans–Jensen III–V	UCHA/PFNA	Retrospective	1, 2, 3, 4, 5, 8, 10, 11, 14, 15, 16
Ucpunar et al. (2019) ( 50 )	31A2/31A3	CHA/PFN	Retrospective	4, 5, 6, 7, 11, 12, 14, 18
Wang et al. (2019) (52)	>31A2.2	CHA/PFNA	Retrospective	4, 8, 11
Wang et al. (2020) ( 51 )	Evans–Jensen III and IV	CHA/PFNA	Retrospective	1, 4, 9, 11, 15
Zhou et al. (2019) ( 53 )	Evans–Jensen III–V	UCHA/PFNA	Observational	3, 4, 8, 10, 11, 13, 14, 17

I, intervention; C, comparison; UCHA, cementless hemiarthroplasty; CHA, cement hemiarthroplasty; PFN, proximal femoral nail; PFNA, proximal femoral nail anti-rotation; NR, not reported; 1, Harris hip score (initial); 2, Harris hip score (6 month); 3, Harris hip score (final); 4, operative time; 5, superficial infection; 6, re-operation; 7, mortality (final); 8, blood loss; 9, ambulation time; 10, full weight-bearing time; 11, hospital stay; 12, mortality (early); 13, bedsores; 14, deep venous thrombosis; 15, general complications; 16, implant-related complications; 17, implant-unrelated complications; 18, blood transfusion.

**Table 2:** The Newcastle–Ottawa scale (NOS) of retrospective studies.

Study	Selection				Comparability	Outcome			Total
	1	2	3	4	5	6	7	8	
Agar et al. (2021) ( 31 )	*	*	*	*	**	*	*	*	9
Cai et al. (2022) ( 32 )	*	*	*	*	**		*	*	8
Canbeyli et al. (2021) ( 33 )	*	*	*	*	*		*	*	7
Çelen and Gazi (2022) (34)	*	*	*	*	*		*	*	7
Çiloğlu et al. (2022) ( 36 )	*	*	*	*	**		*	*	8
Huai-dong (2016) (27)	*	*	*	*	**		*	*	8
Feng et al. (2017) ( 37 )	*	*	*	*	*		*	*	7
Hussain et al. (2017) ( 39 )	*	*	*	*	*		*	*	7
Kilinc and Pazarci (2021) (42)	*	*	*	*	*		*	*	7
Li e et al. (2013) ( 46 )	*	*	*	*	*		*	*	7
Li et al. (2020) ( 45 )	*	*	*	*	**		*	*	8
Ming (2012) (29)	*	*	*	*	*		*	*	7
Liu et al. (2021) ( 47 )	*	*	*	*	*		*	*	7
Chao-Jian (2016) (28)	*	*	*	*	**		*	*	8
Pang et al. (2013) ( 48 )	*	*	*	*	*		*	*	7
Song et al. (2022) ( 49 )	*	*	*	*	**		*	*	8
Ucpunar et al. (2019) ( 50 )	*	*	*	*	*		*	*	7
Wang et al. (2019) (52)	*	*	*	*	*		*	*	7
Wang et al. (2020) ( 51 )	*	*	*	*	*		*	*	7
Zhou et al. (2019) ( 53 )	*	*	*	*	**		*	*	8

1, representativeness of the exposed cohort; 2, selection of the non-exposed cohort; 3, ascertainment of exposure; 4, demonstration that outcome of interest was not present at start of study; 5, comparability of cohorts on the basis of the design or analysis, extra asterisk for study controls for any additional factor; 6, assessment of outcome; 7, follow-up long enough for outcomes to occur; 8, adequacy of follow-up of cohorts; 0–3, very high risk of bias; 4–6, high risk of bias; 7–9, low risk of bias.

### Patient demographics

A total of 2,517 patients were enrolled: 1,265 patients in the HA group and 1,252 patients in the PFN/PFNA group. The majority of studies originated from China (16 studies), followed by Turkey (6 studies), India (4 studies), and South Korea (1 study). Most patients were senile, typically in their 70s–80s. All studies reported statistical insignificance in terms of gender. Patient demographics are summarized in Table 3.

**Table 3:** Demographics of patients.

Study	Region	Follow-up (months)	Age (years)	Gender (male, female)	Sample size
		I/C	I/C	I/C	I/C
Agar et al. (2021) ( 31 )	Turkey	23.3/21.9	82.2 ± 5.7/81.5 ± 7.4	27, 67/21, 56	94/77
Cai et al. (2022) ( 32 )	China	24	82.19 ± 3.96/80.88 ± 4.90	16, 20/16, 18	36/34
Canbeyli et al. ( 2021) ( 33 )	Turkey	12	83.17 ± 9.26/82.21 ± 9.51	NR	84/58
Çelen and Gazi (2022) (34)	Turkey	24.1 ± 11.9	80.0 ± 6.9/77.3 ± 7.3	16, 34/16, 30	52/46
Chen et al. (2017) ( 35 )	China	17	71.23 ± 2.62/72.31 ± 2.96	17, 18/15, 17	35/32
Çiloğlu et al. (2022) ( 36 )	Turkey	24	84.59 ± 7.7/83.89 ± 7.7	30, 45/26, 49	75/75
Huai-dong (2016) (27)	China	12	85 ± 4.4/84 ± 3.6	NR	30/30
Feng et al. (2017) ( 37 )	China	18	80.82 ± 5.92/73.41 ± 7.66	12, 16/10, 14	28/24
Garg et al. (2022) ( 38 )	India	12	76.24 ± 10.94/70.38 ± 7.79	13, 22/10, 25	35/35
Hussain et al. (2017) ( 39 )	India	6	NR	NR	30/30
Jolly et al. (2019) ( 40 )	India	12	78.7 ± 8.3/81.2 ± 7.8	NR	48/46
Joshi et al. (2023) ( 41 )	India	6	73.13 ± 7.37/72.93 ± 7.9	10, 20/12, 18	30/30
Kilinc and Pazarci (2021) (42)	Turkey	20.97 ± 17.47/ 25.41 ± 19.44	82.17 ± 6.29/80.20 ± 5.43	27, 44/21, 25	71/46
Kim et al. (2005) ( 43 )	Korea	36	82 ± 3.4/81 ± 3.2	6, 23/8, 21	29/29
Li e et al. (2013) ( 46 )	China	18	73.6 ± 4.7/72.8 ± 5.8	NR	32/42
Lei (2015) (44)	China	12	76.64 ± 1.32/75.07 ± 1.93	10, 15/11, 14	25/25
Li et al. (2020) ( 45 )	China	18.72 ± 3.49	83.76 ± 6.24/83.20 ± 4.84	14, 32/17, 44	46/61
Ming (2012) (29)	China	12	81.5/85.8	32, 40/36, 42	72/78
Chao-Jian (2016) (28)	China	6	76.8 ± 2.4/77.2 ± 2.8	29, 19/26, 22	48/48
Liu et al. (2021) ( 47 )	China	12	80.3 ± 2.1/80.2 ± 2.1	22, 18/21, 19	40/40
Chao-Jian (2016) (28)	China	12	80.4 ± 5.8/79.7 ± 6.1	15, 48/12, 46	63/58
Pang et al. (2013) ( 48 )	China	12	83.5/84.7	16, 20/18, 21	36/39
Song et al. (2022) ( 49 )	China	12	81.0 ± 9.1/79.9 ± 6.1	9, 21/5, 27	30/32
Ucpunar et al. (2019) ( 50 )	Turkey	12	87 ± 4.1/85.9 ± 4.6	33, 43/19, 45	64/76
Wang et al. (2019) (52)	China	12	77.82 ± 6.76/76.71 ± 7.04	40, 21/38, 26	61/64
Wang et al. (2020) ( 51 )	China	NR	82.9 ± 2.4/84.3 ± 2.9	10, 14/21, 15	24/36
Zhou et al. (2019) ( 53 )	China	36	83.8 ± 6.4/83.5 ± 4.8	27, 20/36, 25	47/61

I, intervention; C, comparison; NR, not reported. Values after ± are standard deviations.

### Outcomes

We divided the outcomes into three divisions: function, complication, and perioperative matters. Function referred to the HHS, ambulation time, and full weight-bearing time. Complication referred to general complications, implant-related complications, implant-unrelated complications, re-operation, bedsores, deep venous thrombosis, and superficial infection. We did not report deep infection because few studies reported it, leading to insufficient data for further synthesis. The remaining six effect sizes were operative time, hospital stay, blood loss, blood transfusion, and mortality in both early and final stages. Details are listed in Supplementary Tables S1–S3. We reported the results in terms of raw mean differences (MDs) and risk ratios, with 95% confidence intervals (CIs) and *p*-values from *Z* tests. We tested heterogeneity using the Cochran Q test, with results reported as *p*-values and *I*^2^. When high *I*^2^ values were detected, we calculated PIs to decide whether to accept or refuse the synthesis results. Subgroup analyses and meta-regression were conducted when appropriate. We ran Egger's test for publication bias and decided on the existence based on data distribution.

### Functional outcomes

#### Harris hip scores (initial)

**Figure 3:**
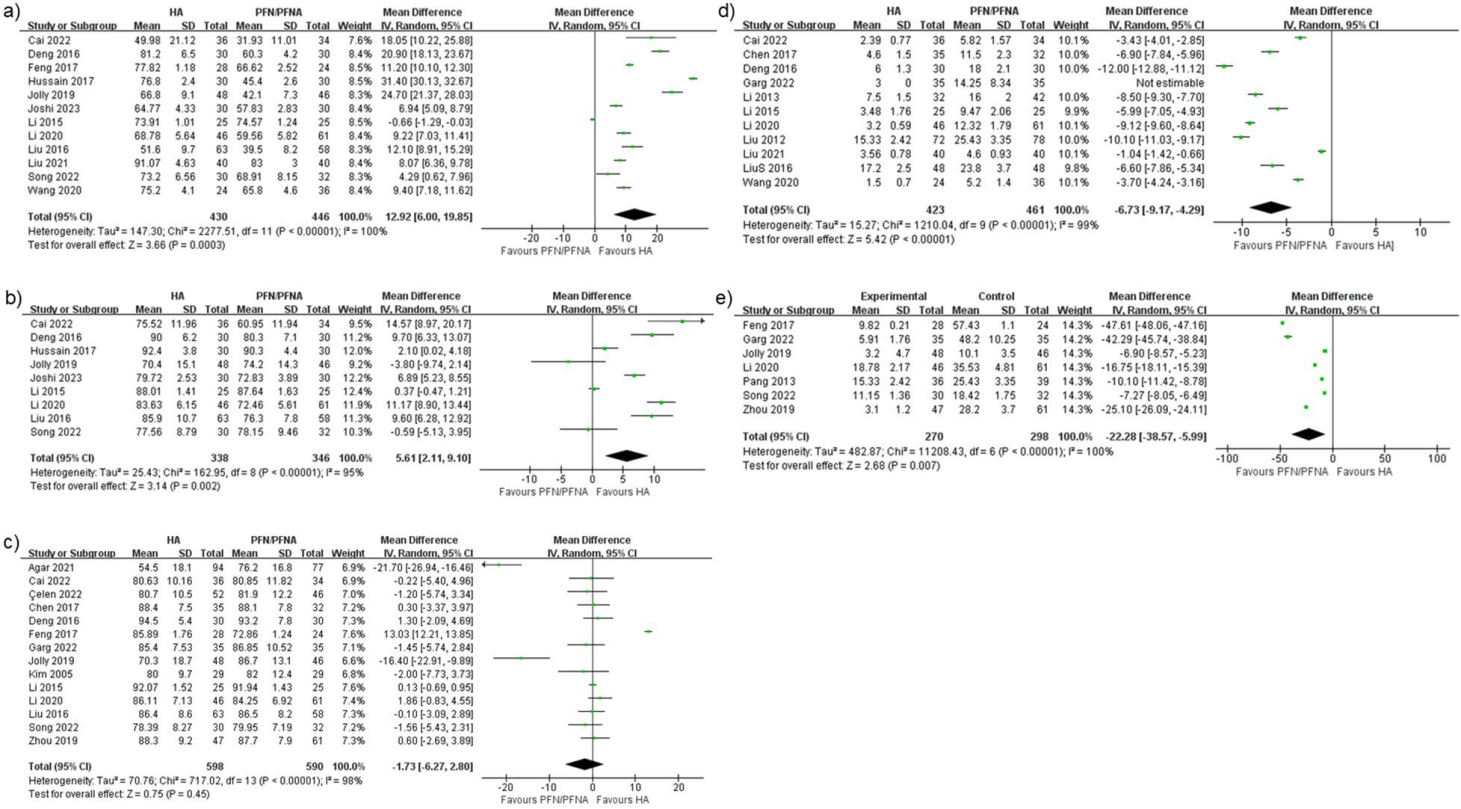
Forest plots of **(a)** Harris hip scores (initial), **(b)** Harris hip scores (6-month), **(c)** Harris hip scores (final), **(d)** ambulation time, and **(e)** full weight-bearing time.

**Figure 4:**
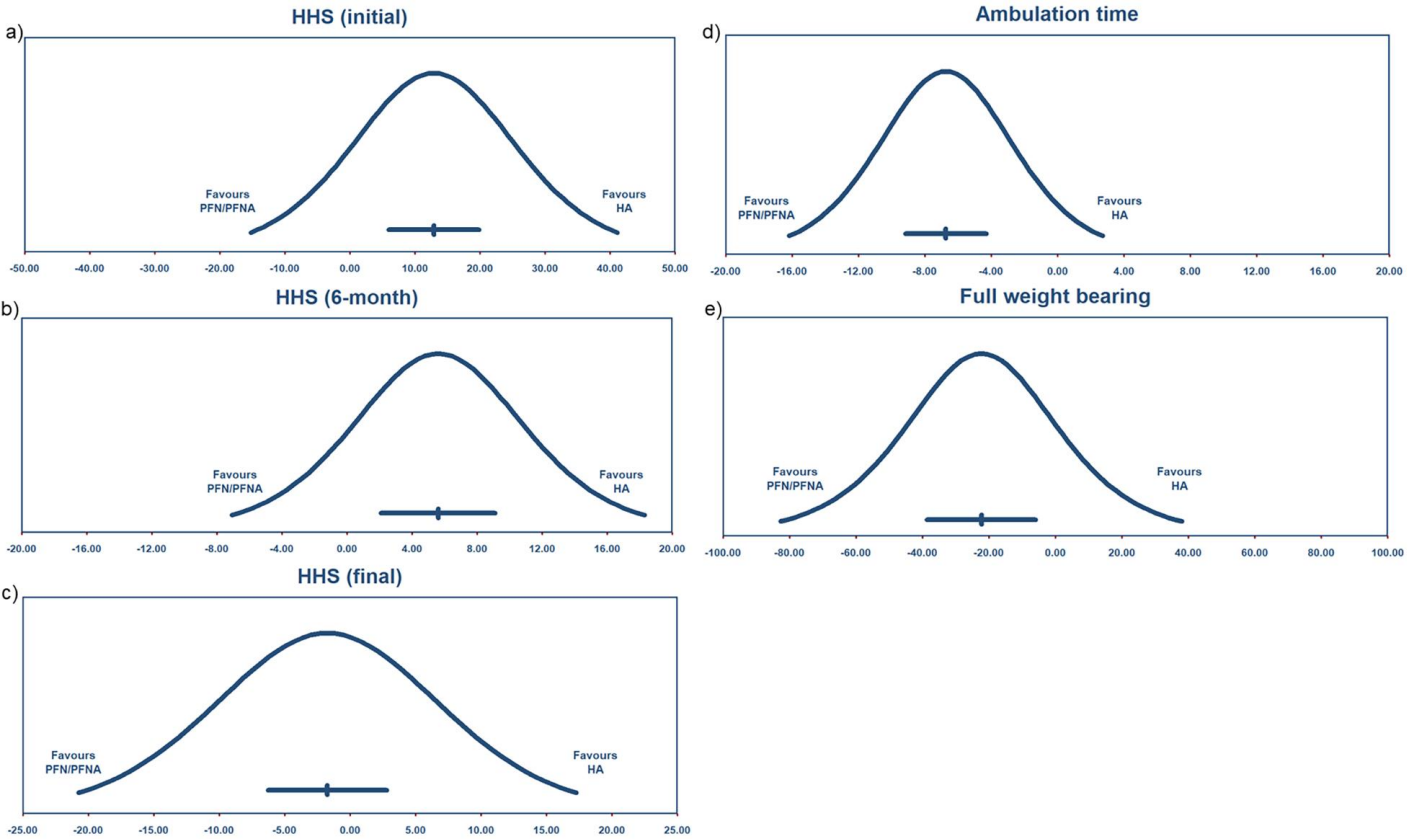
Prediction interval of function results **(a)** HHS (initial), **(b)** HHS (6-month), **(c)** HHS(final), **(d)** ambulation time, and **(e)** full weight bearing.

We defined Harris hip scores within three months after surgery as initial scores. Fourteen studies reported this result, yielding a MD of 12.92 points with a 95% CI (6.00–19.85), as shown in Figure 3a, and the *Z* test indicated *p* < 0.05. Heterogeneity was statistically significant, with *I*^2^ = 100%. The PI was −15.24 to 41.089, as shown in Figure 4a. Egger's test was insignificant with a two-tailed *p*-value of 0.15. The dispersion of PI indicated that HHS (initial) performed better in the HA group despite its high *I*^2^. We conducted subgroup analyses by region and by PFN/PFNA, ran meta-regression of effect sizes by year, and tested heterogeneity stability using the studies-out method. No moderator was found that could reduce the heterogeneity.

#### Harris hip scores (6-month)

The intermediate time—3–6 months after surgery—was analyzed separately as an independent index for function assessment. We detailed the time periods since an overall time estimate may not reveal the true relationship between arthroplasty and internal fixations. The staged HHS performed like pre-subgroup analysis. Nine studies were enrolled, and the mean difference was 5.61 points, with 95% CI (2.11–9.10), as shown in Figure 3b. The *Z* test indicated a significant value, with *p* < 0.05. The heterogeneity rate *I*^2^ was 95%. We calculated a PI of −7.044 to 18.255, as shown in Figure 4b. The dispersion of effect sizes supported a superior outcome for HA compared with PFN/PFNA.

#### Harris hip scores (final)

**Figure 5:**
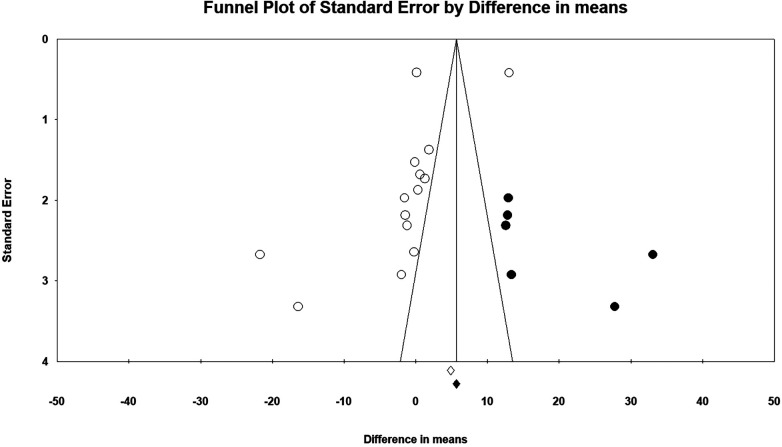
HHS (final) funnel plot.

The final stage of HHS is after 6 months following surgery. The mean difference was −1.73 points with 95% CI (−6.27 to 2.80), but it was not significant with a *p* = 0.45 (Figure 3c). The *I*^2^ was 98%, and the PI ranged from −20.743 to 17.276 points. Therefore, the HA did not show a significantly superior HHS compared with PFN/PFNA. Sensitivity analysis [one-study removal method, Feng et al. (37)] indicated a different result: HHS −2.54, 95% CI (−5.020 to −0.065). We found no factors that might jeopardize the integrity of Feng's report; therefore, exclusion was deemed inappropriate. Egger's test was borderline insignificant with a *p*-value of 0.06 (two-tailed). The funnel plot reflected the potential bias. After Duval and Tweedie's trimming and filling process (Figure 5), the theoretical HHS difference was 4.23497, with 95% CI (0.19982–8.27013). The theoretical result after trimming and filling indicated that the conclusion was not robust, but it should be carefully interpreted. So we deemed this theoretical HHS result only an interpretation of an unrobust result due to failing the sensitivity test, not the clinically meaningful effect size. As such, we upheld the primary result, concluding that it was not robust.

#### Ambulation time

The duration from after surgery to first ambulation was defined as ambulation time. Eleven studies were enrolled (27, 29, 30, 32, 35, 38, 44, 46, 47, 51). The mean difference was −6.73 days, with 95% CI (−9.17 to −4.29), significantly favoring HA; HA patients could ambulate earlier, as shown in Figure 3d. Heterogeneity was significant (*p* < 0.05), with *I*^2^ of 99%. The PI ranged from −16.184 to 2.725 days, with the majority of the dispersion lying to the left of zero, as shown in Figure 4d. Therefore, we considered that the ambulation time was longer in PFN/PFNA patients.

#### Full weight-bearing time

Seven studies reported full weight-bearing time (37, 38, 40, 45, 48, 49, 53). The mean difference was −22.28 days, with 95% CI (−38.57 to −5.99), and it was statistically significant, as shown in Figure 3e. This indicated that HA patients achieved full weight-bearing 22.28 days earlier than PFN/PFNA patients. Heterogeneity was significant (*p* < 0.05), the *I*^2^ was 100%, and no moderator was found for subgroup analysis or regression. Since the PI ranged from −82.669 to 38.117 days (Figure 4e), we were unable to firmly conclude that HA benefited patients more than PFN/PFNA in terms of full weight-bearing time.

### Perioperative condition and mortality

#### Operative time

**Figure 6:**
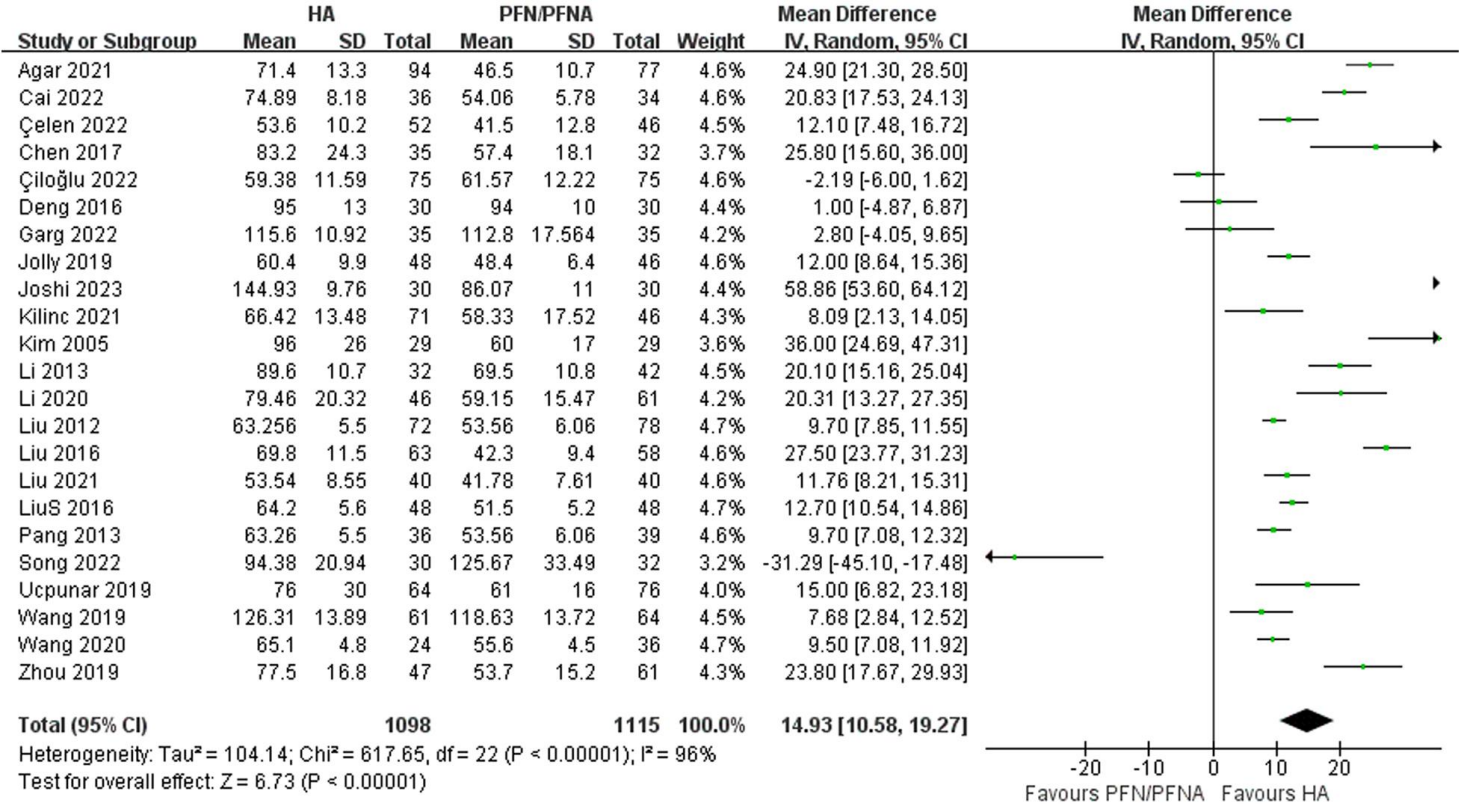
Forest plot of operative time.

**Figure 7:**
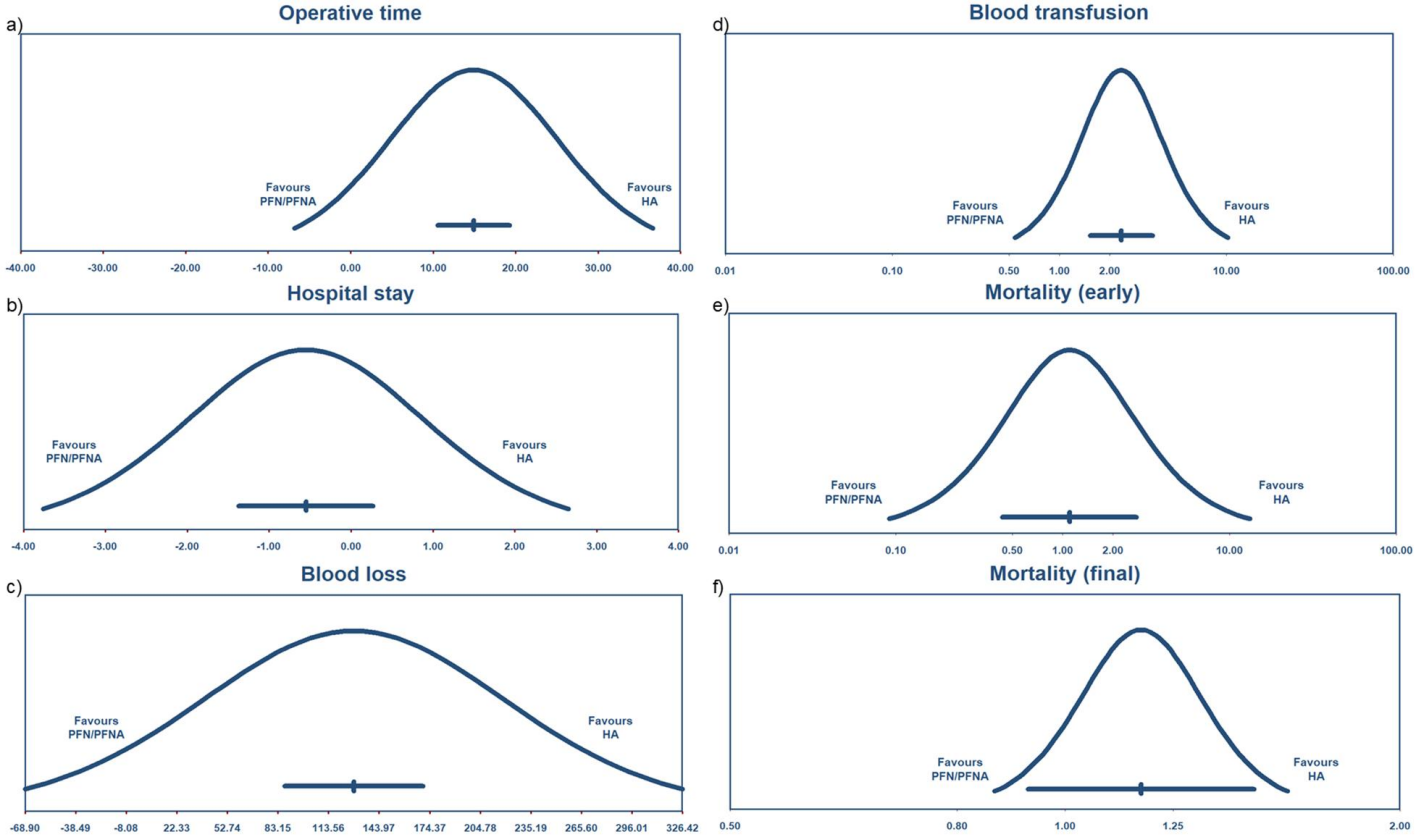
Prediction interval of perioperative condition and mortality **(a)** operative time, **(b)** hospital stay, **(c)** blood loss, **(d)** blood transfusion, **(e)** mortality (early), and **(f)** mortality (final).

Twenty-three studies reported operative time (27–32, 34–36, 38, 40–43, 45–53). The result indicated a significant favoring of PFN/PFNA, with a mean difference of 14.93 min and 95% CI (10.58–19.27), as shown in Figure 6, indicating that HA required a modestly longer surgical duration. The *I*^2^ was 96% and the PI ranged from −6.791 to 36.645 min (Figure 7a). As such, the result demonstrated explicit operative time inequality.

#### Hospital stay

**Figure 8:**
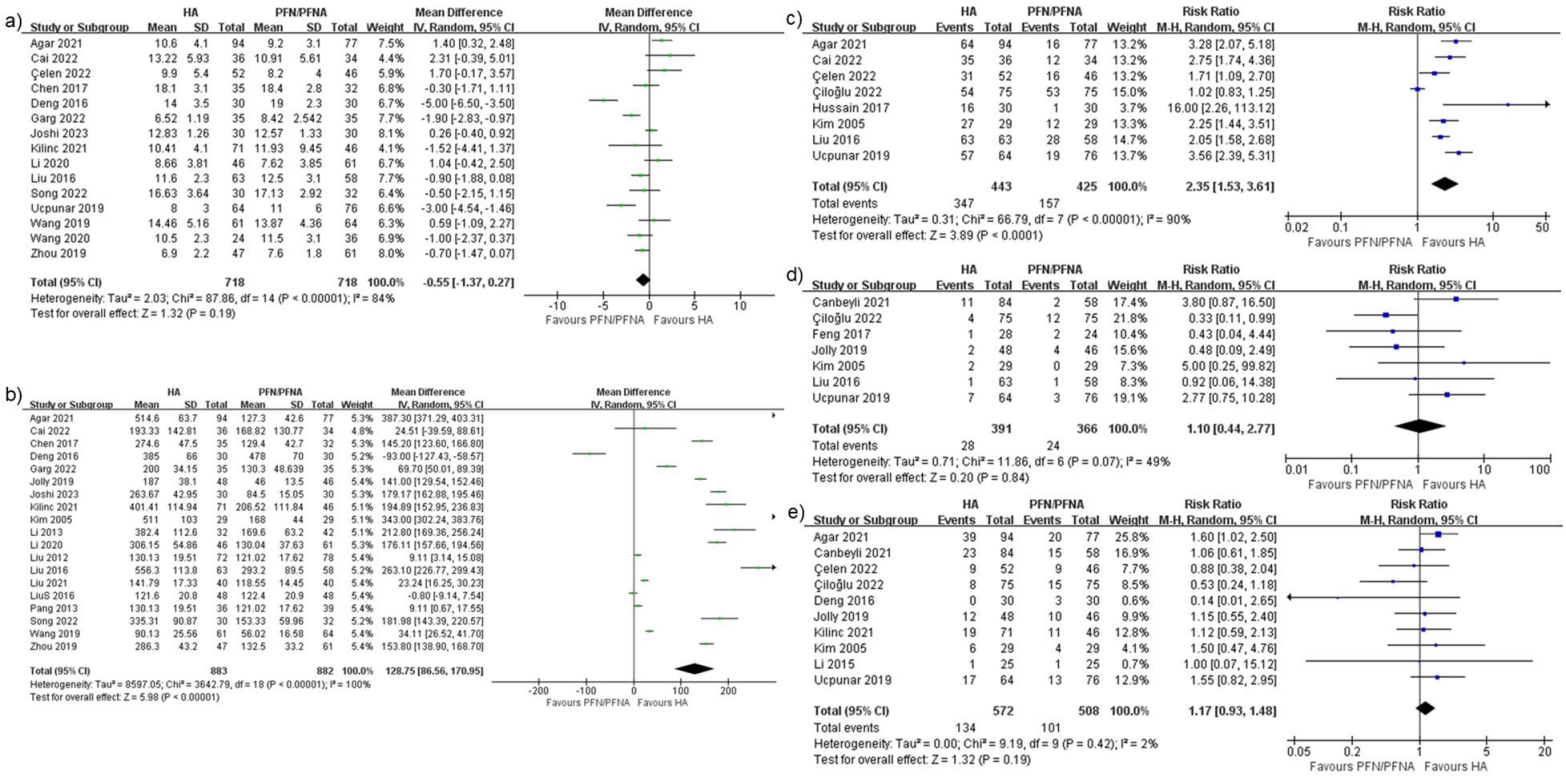
Forest plots of **(a)** Hospital stay, **(b)** Blood loss, **(c)** Blood transfusion, **(d)** Mortality (early), **(e)** Mortality (final).

Fifteen studies (27, 28, 31, 32, 34, 35, 38, 41, 42, 45, 49–53) reported hospital stay or inpatient duration. The mean difference was −0.55 days with 95% CI (−1.37 to 0.27), as shown in Figure 8a. The *I*^2^ was 84%, and the PI ranged from −3.757 to 2.654 days (Figure 7b). Overall, the results indicated that HA and PFN/PFNA were associated with comparable hospital stays, with no significant different observed. No moderators were identified for further analysis.

#### Blood loss

Nineteen studies (27–32, 35, 38, 40–43, 45–49, 52, 53) compared blood loss. The mean difference was 128.75 mL, with 95% CI (86.56–170.95) (Figure 8b). The heterogeneity was considerable, with a high *I*^2^ of 100%. The PI was 86.557–170.945 mL (Figure 7c), supporting the conclusion that the HA process was associated with more blood loss.

#### Blood transfusion

Blood transfusion was a consequential result, mainly due to blood loss or patients’ medical conditions. Eight studies (28, 31, 32, 34, 36, 39, 43, 50) reported blood transfusion, with a risk ratio of 2.35, 95% CI (1.53–3.61), and *I*^2^ of 90% (Figure 8c). The PI was 0.543–10.168 (Figure 7d), indicating that blood transfusion occurred more in patients who underwent the HA procedure.

#### Mortality (early)

We categorized mortality according to duration after surgery: early stage (less than 3 months) and final stage (more than 3 months). We considered that mortality outcomes may differ between these stages. Seven studies (28, 33, 36, 37, 40, 43, 50) reported early mortality, with a risk ratio of 1.10 and 95% CI (0.44–2.77), indicating no statistically significant difference, as shown in Figure 8d. The *I*^2^ was 49% and the PI was 0.092–13.207 (Figure 7e). In conclusion, early mortality did not differ significantly between the HA and PFN/PFNA groups.

#### Mortality (final)

Ten studies (27, 31, 33, 34, 36, 40, 42–44, 50) reported final mortality. The risk ratio was 1.17 with 95% CI (0.93–1.48), as shown in Figure 8e, which was not significant. The *I*^2^ was 2% and the PI was 0.864–1.584 (Figure 7f). In conclusion, there was no solid evidence suggesting that final mortality differed between the HA and PFN/PFNA groups.

### Complications

#### General complications

**Figure 9:**
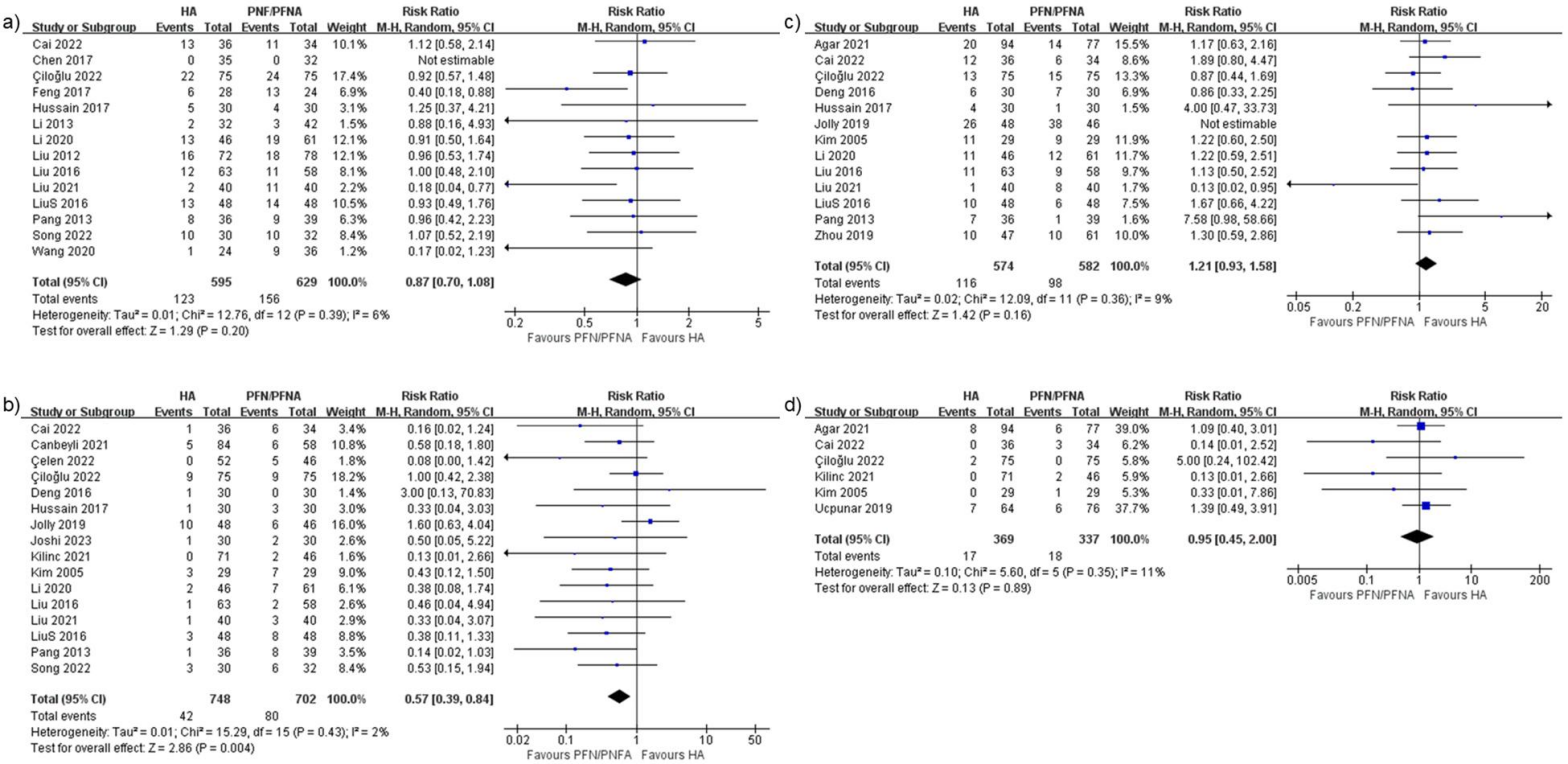
Forest plots of **(a)** general complications, **(b)** implant-related complications, **(c)** implant-unrelated complications, and **(d)** re-operation.

**Figure 10:**
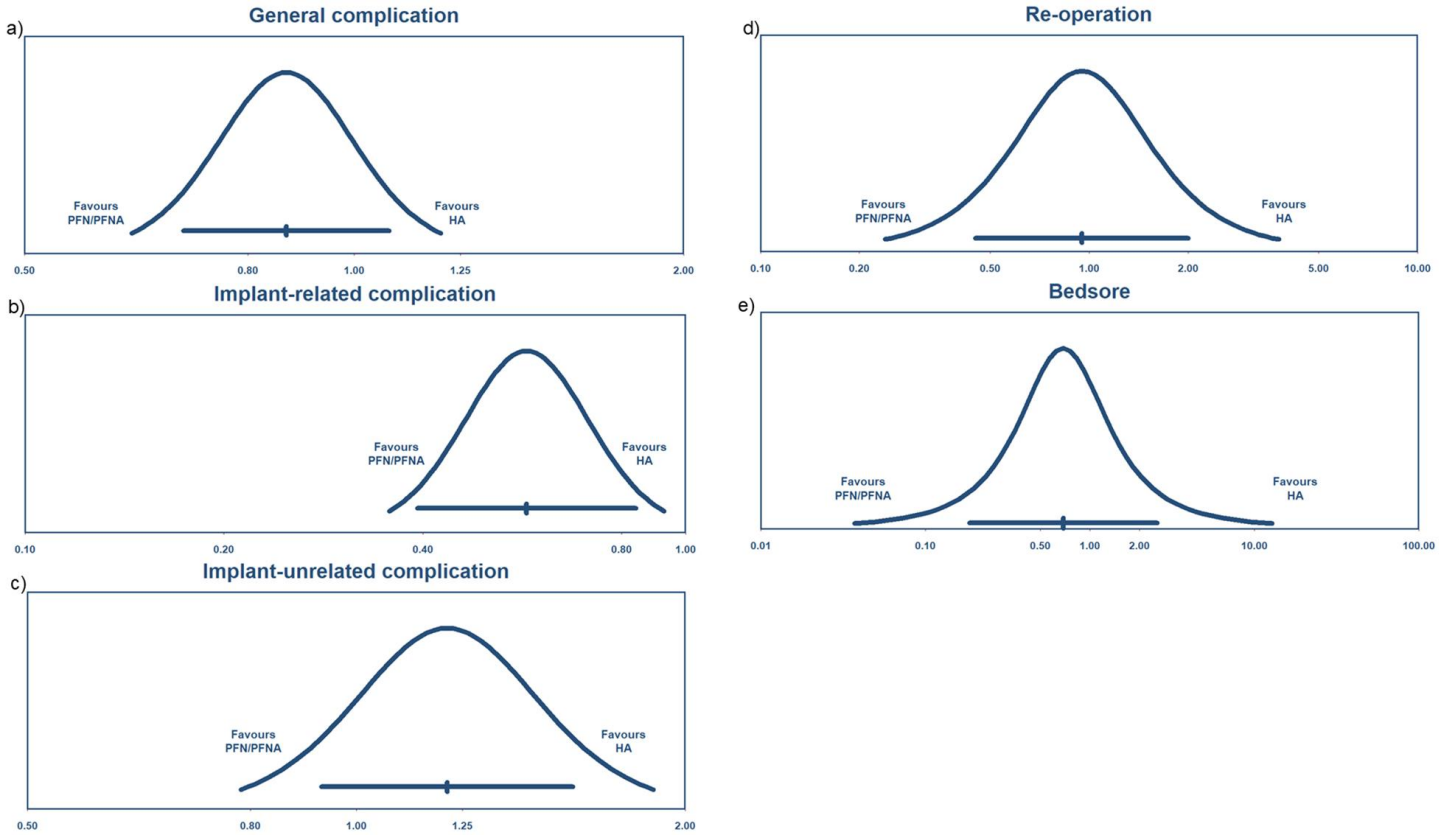
Prediction interval of complications: **(a)** general complications, **(b)** implant-related complications, **(c)** implant-unrelated complications, **(d)** re-operation, and **(e)** bedsore.

Fourteen studies (28–30, 32, 35–37, 39, 45–49, 51) reported general complications, with a risk ratio of 0.87 and 95% CI (0.7–1.08). The heterogeneity rate was 6% (Figure 9a) and the PI was 0.627–1.199 (Figure 10a). These findings indicated that there were no significant differences between the HA and PFN/PFNA groups, in overall complication rates.

#### Implant-related complications

Implants and implant-related complications are the most prominent difference between the HA and PFN/PFNA groups. It is essential to separately investigate the effect sizes. Sixteen studies (27, 28, 30, 32–34, 36, 39–43, 45, 47–49) reported the synthesis risk ratio of 0.57, with 95% CI (0.39–0.84), as shown in Figure 9b. The *I*^2^ was 2% and the PI was 0.356–0.926 (Figure 10b). All data indicated that PFN/PFNA was associated with more implant-related complications than HA.

#### Implant-unrelated complications

Like implant-related complications, implant-unrelated complications also carry implications, particularly in terms of medical interactions as well as direct and indirect effects. Thirteen studies reported complications unrelated to implants. However, one study (40) reported a significantly high complication rate in both the HA (26/48) and PFN/PFNA (38/46) groups. In clinical practice, a surgical device should not be utilized if it causes significant complications in patients, even if the complications are not related to the implant itself. One passable explanation is that individual patients may have suffered from multiple complications—such as, DVT, bedsores, or superficial or deep infections—but no details were provided in the study. As the rate was still too high, this study was excluded. Only 12 studies (27, 28, 30–32, 36, 39, 43, 45, 47, 48, 53) were enrolled, with an insignificant risk ratio of 1.21 and 95% CI (0.93–1.58); the *I*^2^ was 9% (Figure 9c) and the PI was 0.784–1.870 (Figure 10c). As such, there was no evidence indicating that HA differed from PFN/PFNA in implant-unrelated complications.

#### Re-operation

Six studies (31, 32, 36, 42, 43, 50) reported re-operation or revision. Four (32, 36, 42, 43) of these overlapped with the implant-related complication group. The combined risk ratio was 0.95, with 95% CI (0.45–2.00); the *I*^2^ was 11%, as shown in Figure 9d, and the PI was 0.240–3.772 (Figure 10d). In general, no significant statistical difference was detected in re-operation between HA and PFN/PFNA.

#### Bedsores

**Figure 11:**
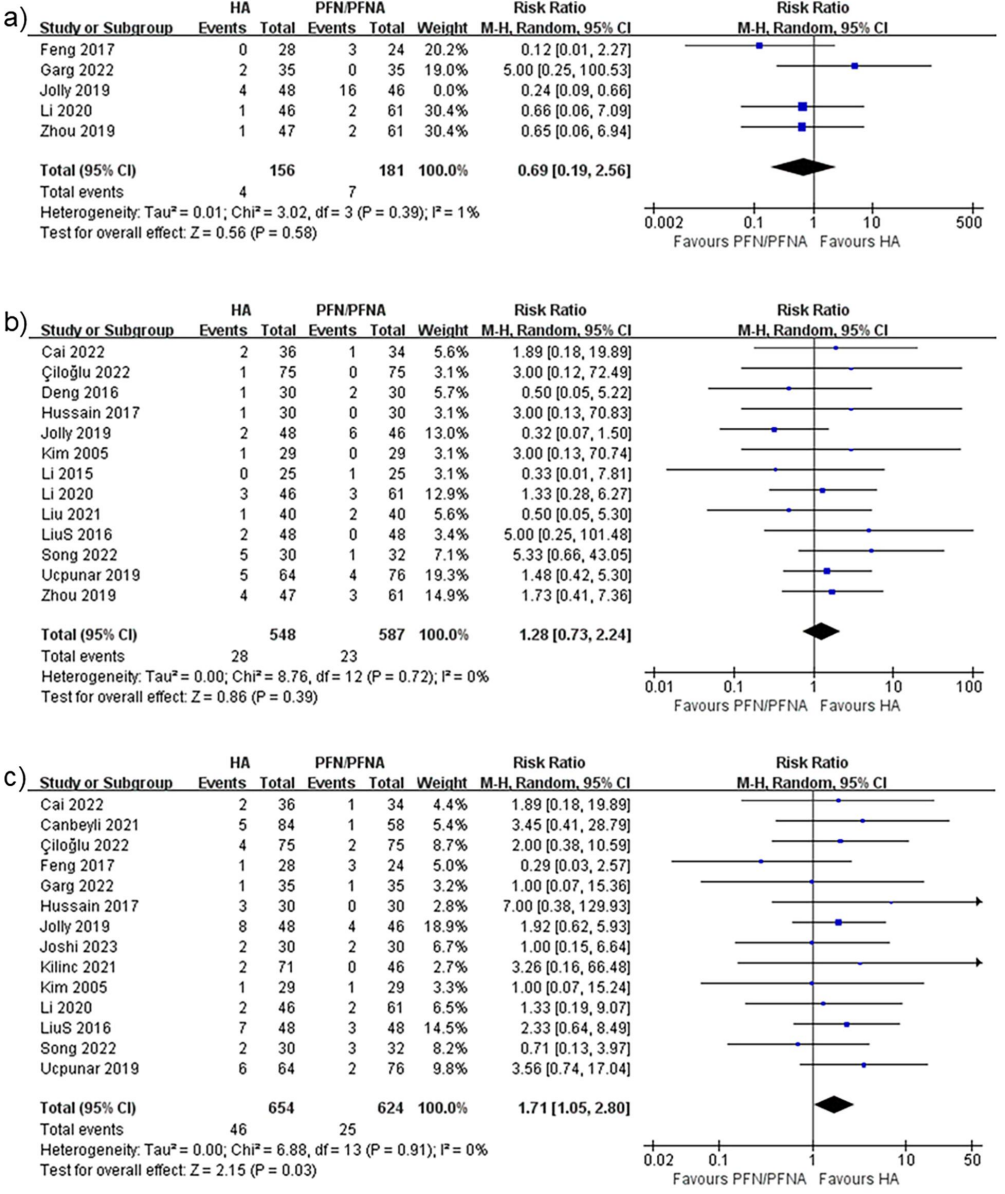
Forest plots of **(a)** bedsores, **(b)** DVT, and **(c)** superficial infection.

Five studies (37, 38, 40, 45, 53) reported the incidence of bedsores. However, Jolly et al. (40) reported an unacceptable PFN/PFNA bedsores rate of 16/46. One previous study (54) estimated that the prevalence of bedsores/pressure ulcers in India was 4.94%, which was similar to that reported in other studies. The approximately 35% bedsore rate seems exceptionally high and disastrous for any nursing system or hospital. We therefore excluded this study from the bedsore analysis. The risk ratio was 0.69, with 95% CI (0.19–2.56) (Figure 11a); the heterogeneity rate was 1% and the PI was 0.185–2.559 (Figure 10e). These findings indicated no statistically significant difference in bedsore comparison between HA and PFN/PFNA.

#### DVT

Thirteen studies reported DVT incidence. The risk ratio was 1.28 with 95% CI (0.73–2.24), as shown in Figure 11b. The heterogeneity was a perfect 0%, and thus no PI was calculated. These findings indicated no statistically significant difference in DVT risk between the two surgical methods.

#### Superficial infection

The final effect size was superficial infection. Although deep infection carries greater clinical weight due to its catastrophic consequences compared with superficial infection, few studies reported it or demonstrated it in a suitable manner. Fourteen studies (30, 32, 33, 36–43, 45, 49, 50) reported superficial infection, with a risk ratio of 1.71, 95% CI (1.05–2.80) (Figure 11c), *I*^2^ equal to 0, and no PI.

### Sensitivity analysis

We performed sensitivity analyses using the one-study removal method. Of the 18 effect sizes examined, only the HHS (final) and superficial infection results failed. The HHS (final) has already been discussed in the Results section. For superficial infection, after the sensitivity analysis, four studies reported significant results, with lower CI limits of 0.995, 0.968, 0.957, and 0.944—findings that contrasted with the overall synthesis. However, given the synthesis CI (1.05–2.80) and the fact that most CI limits clustered closely around 1.00 (one-study removal method), this borderline result was not considered a failure in sensitivity analysis.

In summary, sensitivity analysis indicated that the HHS (final) did not have a robust result like the other domains.

While the primary findings remained stable, sensitivity analysis using the “one-study removal” method revealed that the final Harris hip score lacked the robustness observed in other domains. Moreover, the superficial infection rate exhibited borderline sensitivity in a minority of subsets (e.g., CI lower limits ranging from 0.94 to 0.99). While these marginal fluctuations reflect the inherent variability of these specific parameters, we have treated them with methodological restraint. Consequently, no definitive conclusions are drawn regarding these outcomes in the final summary. This conservative approach ensures that the study's core conclusions are not overshadowed by the inherent instability of subjective or secondary patient-reported metrics.

### Quality assessment

Six RCT studies were assessed using the RoB method and are presented in Figure 2. Overall, they qualified for data merging. However, deficits were detected in all these studies when it came to performance bias and detection bias. Unlike medical cases, blind assessment could not be conducted in certain orthopedic trials. It is impossible to blind patients from operations they undergo, and aspects such as the incision and prognosis instructions will also reveal the surgical approaches. Moreover, Jolly et al. (40) reported unusual results in a few effect sizes, which raised concern regarding a substantial risk of bias. Despite these complications, the RCTs were qualified for data synthesis.

The remaining 21 studies were retrospective or non-randomized studies and were evaluated using the NOS method. Detailed evaluations are presented in Table 2. We deemed levels of risk bias according to the following scores: 0–3, very high risk of bias; 4–6, high risk of bias; and 7–9, low risk of bias. Only Agar et al. (31) achieved a score of nine, being the only study with independent assessment. The other 20 studies all scored between 7 and 9, all ranking at a low risk of bias. In summary, all 27 studies demonstrated essential qualities to support a concrete research process.

### Publication bias

**Figure 12:**
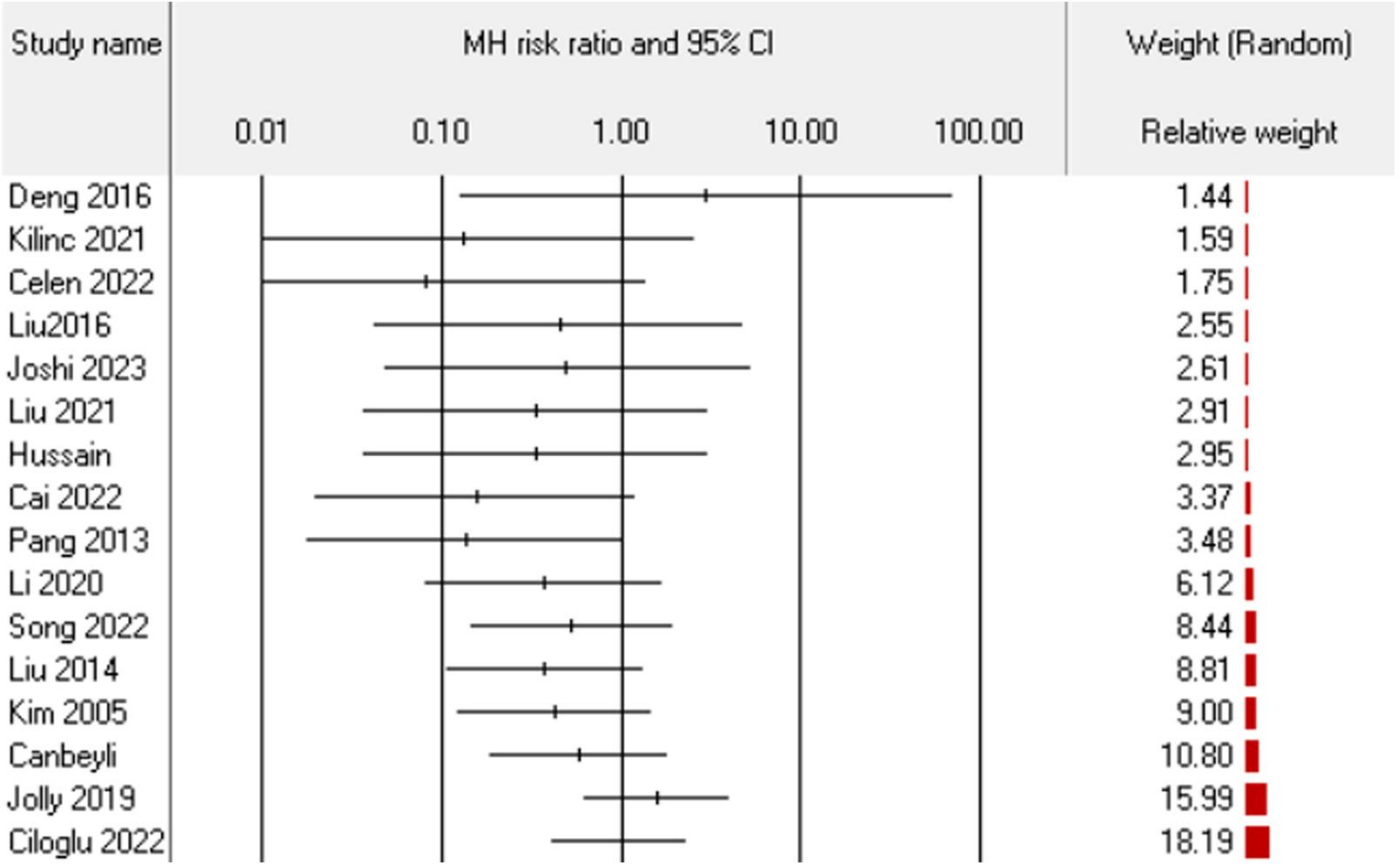
Small-study effect of implant-related complications.

We assessed publication bias using Egger's test across 18 indices/effect sizes. The outcomes of ambulation time, blood loss, blood transfusion, and implant-related complications failed the test, indicating potential publication bias. We analyzed ambulation time, blood loss, and blood transfusion, and all studies in these three domains had similar relative weights, separately. Interestingly, we noted that the implant-related complications group failed the test because of a small-study effect, whereby small studies had larger effect sizes. Egger's publication bias test evaluates the relationship between sample size and effect size using the regression method. In this case, 16 studies had gradually increased weights, as shown in Figure 12. This imbalanced effect size dispersion was attributable to the small-study effect. Furthermore, none of the studies in the implant-related complications group had significant risk ratios, further supporting our opinion that there was no publication bias in this group, unlike the ambulation, blood loss, and blood transfusion groups, which were deemed to have potential publication bias.

### Certainty of evidence

Eighteen effect sizes were assessed using the GRADE evaluation system, with details provided in Supplementary Table S4. In general, the results demonstrated an overall low certainty of evidence because most studies were non-randomized trials/studies. Though there were 6 RCT studies out of the total 27, no effect size items were reported solely from RCTs. For accuracy and reliability, we classified this mixed type as non-randomized, thereby assigning a low level of evidence certainty. No additional factors were identified to upgrade the certainty of evidence. Three items were ranked at a very low level because of potential publication biases. Overall, the whole body of evidence represented an acceptable level of certainty.

## Discussion

For unstable pertrochanteric fractures, many surgeons claim that internal fixation has limited performance and therefore prefer arthroplasty in the event of anticipated device failure. For those who have hip issues or femoral head problems, arthroplasty is a logical choice. However, the removal of a healthy femoral head only due to concerns about the high risk of internal fixation failure is debatable. We conducted this up-to-date, comprehensive systematic review to provide clarity and elaborate results through data and appropriate analyses. Surgeons must distinguish statistical significance from clinical relevance when interpreting these findings. The high statistical heterogeneity observed across the included studies limits the direct translation of these pooled estimates into standardized clinical decision-making. Although high statistical heterogeneity (*I*^2^ > 90%) was noted, it was not pervasive across all analyses as previously suggested. A clear divergence was identified: High *I*^2^ values were confined to the nine domains involving subjective, patient-reported outcomes, whereas the nine objective domains demonstrated minimal heterogeneity (*I*^2^ < 10% or 0%). Given that the directionality of effect remained consistent across all 18 domains—as confirmed by extensive subgroup and meta-regression analyses—the high *I*^2^ in subjective metrics reflects real-world clinical diversity rather than methodological instability. By synthesizing *I*^2^, PI, consistency in effect direction, and Egger's test results, our conclusions remain conservative and data-driven. We intentionally reported these results to remind readers that while objective outcomes are highly stable, the interpretation of subjective, high-heterogeneity results requires appropriate caution.

It is important to note that the PI reflects the range of true variance ($\tau^{2}$). While our PIs were wider than the CIs and crossed the null line, this pattern represents the inherent variability of the treatment effects across different clinical settings. Clinical significance should be interpreted by focusing on the PI's coverage area relative to the horizontal axis (clinical effect size). The fact that the PI spans the line of no effect suggests that while the treatment is effective on average, its real-world application may yield varying results, which serves as a crucial reference for personalized clinical decision-making.

For function comparison, we chose the Harris hip score as an index, and we reported results at consistent stages. In the first 3 months after surgery, the HA group performed better than the PFN/PFNA group, with a difference score of 12.92, which we consider substantial given a total of hundred points on the HHS scale. The HHS scale is mainly composed of five domains: pain, disability, function, deformity, and range of motion. We assume that the HA group will achieve greater early pain relief and experience early withdrawal of the cane or other support devices. Deformity and range of motion were only covered at a maximum of 4 and 5 points, respectively, which would not generate a difference of more than 12.92 points. At the 6-month stage, the HA group remained superior to PFN/PFNA, with a trivial difference of 5.61 points. On a 100-point scale, this is still meaningful. In fact, we will have more solid analysis results if the authors report the HHS in its four domains, i.e., Pain score, Function score, Deformity score, and Range of motion score, which will help us figure out which domain weighs more and matters, but few studies reported this. At the 12-month stage, the data indicated a seemingly insignificant result with publication bias, which means these results should be interpreted with caution with limited weight. Notably, the 12.92 points only revealed statistical significance, and any further clinical interpretation should be made carefully. Over the years, different studies have reported consistent, similar, or even contrasting results regarding functional outcomes measured using the HHS scale. The HHS remains a conventional and effective tool. However, after conducting a clearly specified and comprehensive synthesis of all studies that compare HA and PFN/PFNA to date, publication bias remained, suggesting that authors should use a more appropriate evaluation tool for HA and PFN/PFNA. The ambulation time had publication bias, and the theoretical result after the trim and fill process did not alter the conclusion. In clinical practice, ambulation time is largely determined at the surgeon's discretion. Concern about fixation failure, especially in unstable pertrochanteric fractures, may deter surgeons from aggressive prognosis plans, thus delaying the ambulation time of patients undergoing PFN/PFNA surgery. That may explain the bias, and we suggest treating ambulation time with extreme caution.

The 22.28-day advance proves the superiority of HA, but we have two concerns regarding this. One is that there are no objective guidelines or criteria that define a precise time for surgeons to instruct patients to begin full weight-bearing, because no patient's fracture type and fixation stability are identical. The second concern is that it appears unfair to compare arthroplasty and internal fixation surgery with respect to full weight-bearing time. These aspects contributed to the high *I*^2^. In each study, consistently, HA demonstrated advantages that contributed to faster and better recovery, though the findings were not robust.

Longer operation time is associated with a higher risk to patients. However, whether a discrepancy of approximately 15 min defines that HA is better than PFN/PFNA is debatable. However, we must carefully consider whether a 15 min duration is clinically significant, especially for patients who can tolerate long surgeries.

Hospital stay appeared identical between the two groups. Hospital stay is affected by factors such as patients’ recovery, hospital administrative requests, and patients’ financial considerations. While these issues are important, they might not be consequential.

Blood loss measurement and transfusion requirements were at the discretion of the surgeons. This may explain the presence of high heterogeneity. In general, this difference is not decisive but could influence surgeons’ decision-making.

Mortality is meaningful, particularly as patients are senile or suffering from an extremely high-energy trauma. We categorized mortality into early and final with a 3-month border and detected no discrepancies. Surgeons should consider that both techniques are equivalent in terms of mortality when managing unstable pertrochanteric fractures.

A clear comprehensive scrutiny and explanation of complications can reduce surgeons’ uncertainty when choosing between arthroplasty and internal fixation, which is at the heart of the present debate. In general, the complication rates showed no difference between groups. The heterogeneity rate was as low as 6%, indicated in the results. However, we considered this result insufficient and proceeded with another analysis, separating the complications into the implant-related complications and implant-unrelated complications. Effectively, the separation procedure served as a subgroup analysis, even though the heterogeneity was low. The findings were interesting and meaningful. First, the implant-unrelated complications revealed no difference, which was consistent with the general complications. In an empirical sense, arthroplasty can be expected to give patients earlier and greater range of motion, and this will decrease the incidence of implant-unrelated complications such as bedsores, DVT, and superficial infection. But data did not support this assumption. This 12-study result, with a low *I*^2^ of 9% and an insignificant difference, indicates that surgeons should not take such complications into consideration when making a surgical plan vis-à-vis unstable pertrochanteric fractures. By contrast, implant-related complications—including cut-out, failure, peri-implant fracture or peri-prosthesis fracture, and peri-implant infection—were deemed more closely associated with implant choice. HA only demonstrated a 0.57 times higher risk compared to PFN/PFNA. Although publication bias was detected, it was due to the small-study effect, as discussed earlier. This result is vital. However, we still insist on equivalence between PFN/PFNA and HA. The re-operation rate was not significantly different. Significant implant-related complications that did not require reoperation suggest that the instruments had limited effects on the consequential complications, i.e., those that require reoperation. Incidentally, four of six studies in the re-operation group were also included in the implant-related complication groups, which limited the bias caused by different baselines. Even when excluding the remaining two studies, the conclusion is consistent. We believe that surgeons should not overestimate the effect of complications, since the evidence-based results prove that there is no difference between the two methods for unstable pertrochanteric fractures. According to these results, HA may experience more complications, but they are not severe enough to alter re-operation rates.

We attempted to specify complications for deeper analysis, but only bedsores, DVT, and superficial infection were qualified for merging and analysis. Superficial infection demonstrated a trivial difference, which can be explained by the fact that the HA is more invasive and has a larger approach/incision.

While the certainty of evidence was categorized as “Low”—due to the inherent constraints of surgical research, NRSs, and our stringent inclusion criteria—the consistency of findings across 18 domains and the reported PIs provide a stable basis for these observations. Clinical application of these results should be approached with caution, considering individual patient contexts and the specialized nature of these surgical interventions.

Overall, HA demonstrated advantages, with better early HHS scores, around 100 mL less blood loss and transfusion, a shorter operative duration by nearly 15 min, a lower instrument complication rate that does not lead to more re-operations, and a slightly lower superficial infection rate. General and implant-unrelated complications were comparable between the groups. No mortality difference was detected, either in the early or final stage. None of these represent decisive factors that would persuade an orthopedic surgeon to favor hemiarthroplasty over proximal femoral nail (anti-rotation). This convinces us that surgeons should not interfere with a healthy femoral head in settings of unstable pertrochanteric fracture, unless arthroplasty must be conducted.

This study has several limitations. First, orthopedic surgery trials have inherent constraints, wherein complete blinding of patients and surgeons is extremely difficult to enforce. Patients can easily infer the operation they have undergone based on the incisions and post-operative instructions, and surgeons are likewise aware of the surgery performed. This leads to potential performance bias and detection bias. Second, although we merged a total of 27 studies, the data in several domains may not be sufficient for a solid result. Future studies are needed for a more precise result. Third, conventional evaluation methods or standards, such as the HHS, may not correctly represent the true characteristics of an intervention. A more detailed HHS report or adoption of a more pertinent assessment tool may address this limitation. Furthermore, patients’ baseline characteristics likely influenced treatment allocation, introducing potential indication bias between the hemiarthroplasty and PFN groups.

We systematically evaluated potential sources of bias—including patient frailty, surgeon experience, and implant design—through granular subgroup analyses and meta-regressions across 18 distinct domains. Despite these exhaustive efforts, no single clinical variable emerged as a decisive moderator or confounding factor. This absence of a “statistical fix” for the high *I*^2^ in subjective metrics suggests that this heterogeneity is inherent and diffuse, arising from the cumulative interplay of various clinical protocols rather than a single identifiable source. By reporting PI alongside these results, we provide a transparent representation of this variability, offering a more realistic expectation of treatment effects across diverse clinical settings. More studies and higher-quality data are required in the future for better results. Finally, although most non-RCT studies achieved only a low methodological quality, this is the best level they can attain short of an outstanding performance. We believe that more RCTs will make our findings more convincing.

## Conclusion

Both HA and PFN/PFNA are effective strategies for unstable pertrochanteric fractures. HA demonstrated modest advantages in early functional recovery, a lower incidence of implant-related complications, and better in partial perioperative conditions. But no significant differences were detected in long-term function, general complications, implant-unrelated complications, certain specified complications—such as bedsores and DVT—and re-operation rates due to complications. Any clinical interpretation should be performed carefully because of the inherent limitations in NRS and the high heterogeneity in the subjective domains.

Taken together, we conclude that HA achieves outcomes broadly comparable to PFN/PFNA, with equal effects and similar clinical features. Therefore, surgeons should not favor HA when they encounter an unstable pertrochanteric fracture.

## Data Availability

The original contributions presented in the study are included in the article/Supplementary Material, further inquiries can be directed to the corresponding author.
