# Supplementary material for: Hemiarthroplasty vs. proximal femoral nail fixation in unstable pertrochanteric fractures: an updated systematic review and meta-analysis
Source: Front Surg. 2026 Mar 2;13:1782908. doi: 10.3389/fsurg.2026.1782908 (PMC12989514; doi:10.3389/fsurg.2026.1782908)
Supplement: Supplementary file 1 [file Table1.docx]

**Table S1** Summary of Harris Hip Scores, Ambulation, and Full weight bearing time.

| **Study** | **Harris Hip Scores (initial) (initial)** | | **Harris Hip Scores (6 month) month)** | | **Harris Hip Scores (final) (final)** | | **Ambulation time (day)** | | **Full weight Bearing（day）** | |
| --- | --- | --- | --- | --- | --- | --- | --- | --- | --- | --- |
|  | I | C | I | C | I | C | I | C | I | C |
| Agar 2021 |  |  |  |  | 54.5±18.1 | 76.2±16.8 |  |  |  |  |
| Cai 2022 | 49.98±21.13 | 31.93±11.01 | 75.52±11.96 | 60.95±14.94 | 80.63±10.16 | 80.85±11.82 | 2.39±0.77 | 5.82±1.57 |  |  |
| Canbeyli 2021 |  |  |  |  |  |  |  |  |  |  |
| Çelen 2022 |  |  |  |  | 80.7±10.5 | 81.9±12.2 |  |  |  |  |
| Chen 2017 |  |  |  |  | 88.4±7.5 | 88.1±7.8 | 4.6±1.5 | 11.5±2.3 |  |  |
| Çiloğlu 2022 |  |  |  |  | Figure | Figure |  |  |  |  |
| Deng 2016 | 81.2±6.5 | 60.3±4.2 | 90.6±6.2 | 80.3±7.1 | 94.5±5.4 | 93.2±7.8 | 6.0±1.3 | 18.0±2.1 |  |  |
| Feng 2017 | 77.82±1.18 | 66.62±2.52 |  |  | 85.89±1.76 | 72.86±1.24 |  |  | 9.82±0.21 | 57.43±1.10 |
| Garg 2022 |  |  |  |  | 85.40±7.53 | 86.85±10.52 | 3 | 14.25±8.34 | 5.91±1.76 | 48.20±10.25 |
| Hussain 2017 | 76.8±2.4 | 45.4±2.6 | 92.4±3.8 | 90.3±4.4 |  |  |  |  |  |  |
| Jolly 2019 | 66.8±9.1 | 42.1±7.3 | 70.4±15.1 | 74.2±14.3 | 70.3±18.7 | 86.7±13.1 |  |  | 3.2±4.7 | 10.1±3.5 |
| Joshi 2023 | 64.77±4.33 | 57.83±2.83 | 79.72±2.53 | 72.83±3.89 |  |  |  |  |  |  |
| Kilinc 2021 |  |  |  |  |  |  |  |  |  |  |
| Kim 2005 |  |  |  |  | 80±9.7 | 82±12.4 |  |  |  |  |
| Li 2013 |  |  |  |  |  |  | 7.5±1.5 | 16±2 |  |  |
| Li 2015 | 73.91±1.01 | 74.57±1.24 | 88.01±1.41 | 87.64±1.63 | 92.07±1.52 | 91.94±1.43 | 3.48±1.76 | 9.4 ±2.06 |  |  |
| Li 2020 | 68.78±5.64 | 59.56±5.82 | 83.63±6.15 | 72.46±5.61 | 86.11±7.13 | 84.25±6.92 | 3.20±0.59 | 12.32±1.79 | 18.78±2.17 | 35.53±4.81 |
| Liu 2012 |  |  |  |  |  |  | 15.33±2.42 | 25.43±3.35 |  |  |
| LiuS 2016 |  |  |  |  |  |  | 17.2±2.5 | 23.8±3.7 |  |  |
| Liu 2021 | 91.07±4.63 | 83.00±3.00 |  |  |  |  | 3.56±0.78 | 4.60±0.93 |  |  |
| Liu 2016 | 51.6±9.7 | 39.5±8.2 | 85.9±10.7 | 76.3±7.8 | 86.4±8.6 | 86.5±8.2 |  |  |  |  |
| Pang 2013 |  |  |  |  |  |  |  |  | 15.33±2.42 | 25.43±3.35 |
| Song 2022 | 73.20±6.56 | 68.91±8.15 | 77.56±8.79 | 78.15±9.46 | 78.39±8.27 | 79.95±7.19 |  |  | 11.15±1.36 | 18.42±1.75 |
| Ucpunar 2019 |  |  |  |  |  |  |  |  |  |  |
| Wang 2019 |  |  |  |  |  |  |  |  |  |  |
| Wang 2020 | 75.2±4.1 | 65.8±4.6 |  |  |  |  | 1.5±0.7 | 5.2±1.4 |  |  |
| Zhou 2019 |  |  |  |  | 88.3±9.2 | 87.7±7.9 |  |  | 3.1±1.2 | 28.2±3.7 |

**Note.** I intervention; C comparison; initial <3 months; final >1 year.
