# Supplementary material for: Hemiarthroplasty vs. proximal femoral nail fixation in unstable pertrochanteric fractures: an updated systematic review and meta-analysis
Source: Front Surg. 2026 Mar 2;13:1782908. doi: 10.3389/fsurg.2026.1782908 (PMC12989514; doi:10.3389/fsurg.2026.1782908)
Supplement: Supplementary file 3 [file Table3.docx]

**Table S2** Summary of perioperative condition and mortality

| **Study** | **Operative Time** | | **Hospital Stay (day)** | | **Blood Loss (ml)** | | **Blood transfusion** | | **Mortality (early)** | | **Mortality (final)** | |
| --- | --- | --- | --- | --- | --- | --- | --- | --- | --- | --- | --- | --- |
|  | I | C | I | C | I | C | I | C | I | C | I | C |
| Agar 2021 | 71.4±13.3 | 46.5±10.7 | 10.6±4.1 | 9.2±3.1 | 514.6±63.7 | 127.3±42.6 | 64 | 16 |  |  | 39 | 20 |
| Cai 2022 | 74.89±8.18 | 54.06±5.78 | 13.22±5.93 | 10.91±5.61 | 193.33±142.81 | 168.82±130.77 | 35 | 12 |  |  |  |  |
| Canbeyli 2021 |  |  |  |  |  |  |  |  | 11 | 2 | 23 | 15 |
| Çelen 2022 | 53.6±10.2 | 41.5±12.8 | 9.9±5.4 | 8.2±4.0 |  |  | 31 | 16 |  |  | 9 | 9 |
| Chen 2017 | 83.2±24.3 | 57.4±18.1 | 18.1±3.1 | 18.4±2.8 | 274.6±47.5 | 129.4±42.7 |  |  |  |  |  |  |
| Çiloğlu 2022 | 59.38±11.59 | 61.57±12.22 |  |  |  |  | 54 | 53 | 4 | 12 | 8 | 15 |
| Deng 2016 | 95±13 | 94±10 | 14.0±3.5 | 19.0±2.3 | 385±66 | 478±70 |  |  |  |  | 0 | 3 |
| Feng 2017 |  |  |  |  |  |  |  |  | 1 | 2 |  |  |
| Garg 2022 | 115.6±10.92 | 112.80±17.564 | 6.52±1.19 | 8.42±2.542 | 200±34.15 | 130.3±48.639 |  |  |  |  |  |  |
| Hussain 2017 |  |  |  |  |  |  | 16 | 1 |  |  |  |  |
| Jolly 2019 | 60.4±9.9 | 48.4±6.4 |  |  | 187±38.1 | 46±13.5 |  |  | 2 | 4 | 12 | 10 |
| Joshi 2023 | 144.93±9.76 | 86.07±11 | 12.83±1.26 | 12.57±1.33 | 263.67±42.95 | 84.5±15.05 |  |  |  |  |  |  |
| Kilinc 2021 | 66.42±13.48 | 58.33±17.52 | 10.41±4.10 | 11.93±9.45 | 401.41±114.94 | 206.52±111.84 |  |  |  |  | 19 | 11 |
| Kim 2005 | 96±26 | 60±17 |  |  | 511±103 | 168±44 | 27 | 12 | 2 | 0 | 14 | 5 |
| Li 2013 | 89.6±10.7 | 69.5±10.8 |  |  | 382.4±112.6 | 169.6±63.2 |  |  |  |  |  |  |
| Li 2015 |  |  |  |  |  |  |  |  |  |  | 1 | 1 |
| Li 2020 | 79.46±20.32 | 59.15±15.47 | 8.66±3.81 | 7.62±3.85 | 306.15±54.86 | 130.04±37.63 |  |  |  |  |  |  |
| Liu 2012 | 63.256±5.50 | 53.56±6.06 |  |  | 130.13±19.51 | 121.02±17.62 |  |  |  |  |  |  |
| LiuS 2016 | 64.2±5.6 | 51.5±5.2 |  |  | 121.6±20.8 | 122.4±20.9 |  |  |  |  |  |  |
| Liu 2021 | 53.54±8.55 | 41.78±7.61 |  |  | 141.79±17.33 | 118.55±14.45 |  |  |  |  |  |  |
| Liu 2016 | 69.8±11.5 | 42.3±9.4 | 11.6±2.3 | 12.5±3.1 | 556.3±113.8 | 293.2±89.5 | 63 | 28 | 1 | 1 |  |  |
| Pang 2013 | 63.26±5.5 | 53.56±6.06 |  |  | 130.13±19.51 | 121.02±17.62 |  |  |  |  |  |  |
| Song 2022 | 94.38±20.94 | 125.67±33.49 | 16.63±3.64 | 17.13±2.92 | 335.31±90.87 | 153.33±59.96 |  |  |  |  |  |  |
| Ucpunar | 76±30 | 61±16 | 8±3 | 11±6 |  |  | 57 | 19 | 7 | 3 | 17 | 13 |
| 2019 |  |  |  |  |  |  |  |  |  |  |  |  |
| Wang 2019 | 126.31±13.89 | 118.63±13.72 | 14.46±5.16 | 13.87±4.36 | 90.13±25.56 | 56.02±16.58 |  |  |  |  |  |  |
| Wang 2020 | 65.1±4.8 | 55.6±4.5 | 10.5±2.3 | 11.5±3.1 |  |  |  |  |  |  |  |  |
| Zhou 2019 | 77.5±16.8 | 53.7±15.2 | 6.9±2.2 | 7.6±1.8 | 286.3±43.2 | 132.5±33.2 |  |  |  |  |  |  |

**Note.** I intervention; C comparison; initial <3 months; final >1 year.
