# Supplementary material for: Hemiarthroplasty vs. proximal femoral nail fixation in unstable pertrochanteric fractures: an updated systematic review and meta-analysis
Source: Front Surg. 2026 Mar 2;13:1782908. doi: 10.3389/fsurg.2026.1782908 (PMC12989514; doi:10.3389/fsurg.2026.1782908)
Supplement: Supplementary file 4 [file Table4.docx]

**Table S3** Summary of Complications

| **Study** | **GC** | | **IRC** | | **IURC** | | **Re-Operation** | | **Bedsore** | | **DVT** | | **Superficial Infection** | |
| --- | --- | --- | --- | --- | --- | --- | --- | --- | --- | --- | --- | --- | --- | --- |
|  | I | C | I | C | I | C | I | C | I | C | I | C | I | C |
| Agar 2021 |  |  |  |  | 20 | 14 | 8 | 6 |  |  |  |  |  |  |
| Cai 2022 | 13 | 11 | 1 | 5 | 12 | 6 | 0 | 3 |  |  | 2 | 1 | 2 | 1 |
| Canbeyli 2021 |  |  | 5 | 6 |  |  |  |  |  |  |  |  | 5 | 1 |
| Çelen 2022 |  |  | 0 | 5 |  |  |  |  |  |  |  |  |  |  |
| Chen 2017 | 0 | 0 |  |  |  |  |  |  |  |  |  |  |  |  |
| Çiloğlu 2022 | 22 | 24 | 9 | 9 | 13 | 15 | 2 | 0 |  |  | 1 | 0 | 4 | 2 |
| Deng 2016 |  |  | 1 | 0 | 6 | 7 |  |  |  |  | 1 | 2 |  |  |
| Feng 2017 | 6 | 13 |  |  |  |  |  |  | 0 | 3 |  |  | 1 | 3 |
| Garg 2022 |  |  |  |  |  |  |  |  | 2 | 0 |  |  | 1 | 1 |
| Hussain 2017 | 5 | 4 | 1 | 3 | 4 | 1 |  |  |  |  | 1 | 0 | 3 | 0 |
| Jolly 2019 |  |  | 10 | 6 | 26 | 38 |  |  | 4 | 16 | 2 | 6 | 8 | 4 |
| Joshi 2023 |  |  | 1 | 2 |  |  |  |  |  |  |  |  | 2 | 2 |
| Kilinc 2021 |  |  | 0 | 2 |  |  | 0 | 2 |  |  |  |  | 2 | 0 |
| Kim 2005 |  |  | 3 | 7 | 11 | 9 | 0 | 1 |  |  | 1 | 0 | 1 | 1 |
| Li 2013 | 2 | 3 |  |  |  |  |  |  |  |  |  |  |  |  |
| Li 2015 |  |  |  |  |  |  |  |  |  |  | 0 | 1 |  |  |
| Li 2020 | 13 | 19 | 2 | 7 | 11 | 12 |  |  | 1 | 2 | 3 | 3 | 2 | 2 |
| Liu 2012 | 16 | 18 |  |  |  |  |  |  |  |  |  |  |  |  |
| LiuS 2016 | 13 | 14 | 3 | 8 | 10 | 6 |  |  |  |  | 2 | 0 | 7 | 3 |
| Liu 2021 | 2 | 11 | 1 | 3 | 1 | 8 |  |  |  |  | 1 | 2 |  |  |
| Liu 2016 | 12 | 11 | 1 | 2 | 11 | 9 |  |  |  |  |  |  |  |  |
| Pang 2013 | 8 | 9 | 1 | 8 | 7 | 1 |  |  |  |  |  |  |  |  |
| Song 2022 | 10 | 10 | 3 | 6 |  |  |  |  |  |  | 5 | 1 | 2 | 3 |
| Ucpunar 2019 |  |  |  |  |  |  | 7 | 6 |  |  | 5 | 4 | 6 | 2 |
| Wang 2019 |  |  |  |  |  |  |  |  |  |  |  |  |  |  |
| Wang 2020 | 1 | 9 |  |  |  |  |  |  |  |  |  |  |  |  |
| Zhou 2019 |  |  |  |  | 10 | 10 |  |  | 1 | 2 | 4 | 3 |  |  |

**Note.** I intervention, C comparison, GC general complication, IRC implant-related complication, IURC implant-unrelated complication, DVT deep venous thrombosis.
