# Supplementary material for: Hemiarthroplasty vs. proximal femoral nail fixation in unstable pertrochanteric fractures: an updated systematic review and meta-analysis
Source: Front Surg. 2026 Mar 2;13:1782908. doi: 10.3389/fsurg.2026.1782908 (PMC12989514; doi:10.3389/fsurg.2026.1782908)
Supplement: Supplementary file 5 [file Supplementaryfile1.docx]

**PubMed**

((("hip"[MeSH Terms] OR "hip"[All Fields]) AND ("arthroplasty"[MeSH Terms] OR "arthroplasty"[All Fields] OR "arthroplasties"[All Fields])) OR ("arthroplasty, replacement, hip"[MeSH Terms] OR ("arthroplasty"[All Fields] AND "replacement"[All Fields] AND "hip"[All Fields]) OR "hip replacement arthroplasty"[All Fields] OR ("total"[All Fields] AND "hip"[All Fields] AND "arthroplasty"[All Fields]) OR "total hip arthroplasty"[All Fields]) OR ("hemiarthroplasty"[MeSH Terms] OR "hemiarthroplasty"[All Fields] OR "hemiarthroplasties"[All Fields]) OR ("arthroplasty, replacement, hip"[MeSH Terms] OR ("arthroplasty"[All Fields] AND "replacement"[All Fields] AND "hip"[All Fields]) OR "hip replacement arthroplasty"[All Fields] OR ("total"[All Fields] AND "hip"[All Fields] AND "arthroplasty"[All Fields]) OR "total hip arthroplasty"[All Fields]) OR "THA"[All Fields] OR "HA"[All Fields] OR ("arthroplasty, replacement, hip"[MeSH Terms] OR ("arthroplasty"[All Fields] AND "replacement"[All Fields] AND "hip"[All Fields]) OR "hip replacement arthroplasty"[All Fields] OR ("total"[All Fields] AND "hip"[All Fields] AND "replacement"[All Fields]) OR "total hip replacement"[All Fields])) AND ((("proximal"[All Fields] OR "proximalization"[All Fields] OR "proximalize"[All Fields] OR "proximalized"[All Fields] OR "proximalizes"[All Fields] OR "proximalizing"[All Fields] OR "proximally"[All Fields] OR "proximals"[All Fields]) AND ("femor"[All Fields] OR "femorals"[All Fields] OR "femur"[MeSH Terms] OR "femur"[All Fields] OR "femoral"[All Fields]) AND ("nails"[MeSH Terms] OR "nails"[All Fields] OR "nail"[All Fields]) AND ("antirotation"[All Fields] OR "antirotational"[All Fields])) OR (("proximal"[All Fields] OR "proximalization"[All Fields] OR "proximalize"[All Fields] OR "proximalized"[All Fields] OR "proximalizes"[All Fields] OR "proximalizing"[All Fields] OR "proximally"[All Fields] OR "proximals"[All Fields]) AND ("femur"[MeSH Terms] OR "femur"[All Fields] OR "femurs"[All Fields] OR "femur s"[All Fields] OR "femural"[All Fields] OR "femure"[All Fields]) AND ("nails"[MeSH Terms] OR "nails"[All Fields] OR "nail"[All Fields]) AND ("antirotation"[All Fields] OR "antirotational"[All Fields])) OR (("proximal"[All Fields] OR "proximalization"[All Fields] OR "proximalize"[All Fields] OR "proximalized"[All Fields] OR "proximalizes"[All Fields] OR "proximalizing"[All Fields] OR "proximally"[All Fields] OR "proximals"[All Fields]) AND ("femor"[All Fields] OR "femorals"[All Fields] OR "femur"[MeSH Terms] OR "femur"[All Fields] OR "femoral"[All Fields]) AND ("nails"[MeSH Terms] OR "nails"[All Fields] OR "nail"[All Fields])) OR (("proximal"[All Fields] OR "proximalization"[All Fields] OR "proximalize"[All Fields] OR "proximalized"[All Fields] OR "proximalizes"[All Fields] OR "proximalizing"[All Fields] OR "proximally"[All Fields] OR "proximals"[All Fields]) AND ("femur"[MeSH Terms] OR "femur"[All Fields] OR "femurs"[All Fields] OR "femur s"[All Fields] OR "femural"[All Fields] OR "femure"[All Fields]) AND ("nails"[MeSH Terms] OR "nails"[All Fields] OR "nail"[All Fields])) OR "PFNA"[All Fields] OR "PFN"[All Fields] OR ("intramedullary"[All Fields] AND ("nails"[MeSH Terms] OR "nails"[All Fields] OR "nail"[All Fields])) OR ("cephalomedullary"[All Fields] AND ("nails"[MeSH Terms] OR "nails"[All Fields] OR "nail"[All Fields]))) AND ((("unstability"[All Fields] OR "unstable"[All Fields]) AND ("hip fractures"[MeSH Terms] OR ("hip"[All Fields] AND "fractures"[All Fields]) OR "hip fractures"[All Fields] OR ("intertrochanteric"[All Fields] AND "fracture"[All Fields]) OR "intertrochanteric fracture"[All Fields])) OR (("unstability"[All Fields] OR "unstable"[All Fields]) AND ("pertrochanteral"[All Fields] OR "pertrochanteric"[All Fields]) AND ("fractur"[All Fields] OR "fractural"[All Fields] OR "fracture s"[All Fields] OR "fractures, bone"[MeSH Terms] OR ("fractures"[All Fields] AND "bone"[All Fields]) OR "bone fractures"[All Fields] OR "fracture"[All Fields] OR "fractured"[All Fields] OR "fractures"[All Fields] OR "fracturing"[All Fields])) OR (("unstability"[All Fields] OR "unstable"[All Fields]) AND ("hip"[MeSH Terms] OR "hip"[All Fields]) AND "extracapusular"[All Fields] AND ("fractur"[All Fields] OR "fractural"[All Fields] OR "fracture s"[All Fields] OR "fractures, bone"[MeSH Terms] OR ("fractures"[All Fields] AND "bone"[All Fields]) OR "bone fractures"[All Fields] OR "fracture"[All Fields] OR "fractured"[All Fields] OR "fractures"[All Fields] OR "fracturing"[All Fields])) OR "31A2"[All Fields] OR "31A3"[All Fields])

**Web of Science**

((((((TS=(unstable intertrochanteric fracture)) OR TS=(unstable pertrochanteric fracture)) OR TS=(unstable extracapsular fracture)) OR TS=(unstable hip fracture)) OR TS=(31A2)) OR TS=(31A3) and Preprint Citation Index (Exclude – Database)) AND ((((((((TS=(proximal femoral nail antirotation)) OR TS=(proximal femur nail antirotation)) OR TS=(proximal femoral nail)) OR TS=(proximal femur nail)) OR TS=(PFNA)) OR TS=(PFN)) OR TS=(intramedullary nail )) OR TS=(cephalomedullary nail) and Preprint Citation Index (Exclude – Database)) AND ((((((TS=(hip arthroplasty )) OR TS=(total hip arthroplasty)) OR TS=(hemiarthroplasty)) OR TS=(THA)) OR TS=(HA)) OR TS=(total hip replacement) and Preprint Citation Index (Exclude – Database))

**Embase**

‘'hip arthroplasty' OR 'total hip arthroplasty' OR 'hemiarthroplasty' OR 'tha' OR 'ha' OR 'total hip replacement'’ AND ‘'unstable intertrochanteric fracture' OR 'unstable pertrochanteric fracture' OR 'unstable extracapuslar fracture' OR 'unstable hip fracture' OR '31a2' OR '31a3'’ AND ‘'hip arthroplasty' OR 'total hip arthroplasty' OR 'hemiarthroplasty' OR 'tha' OR 'ha' OR 'total hip replacement'’

**Europe-PMC**

("unstable intertrochanteric fracture" OR "unstable pertrochanteric fracture" OR "unstable extracapsular fracture" OR "unstable hip fracture" OR "31A2" OR "31A3" AND "proximal femoral nail" OR "proximal femur nail" OR "proximal femoral nail antirotation" OR "proximal femur nail antirotaiton" OR "intramedullary nail" OR "cephalomedullary nail" AND "hip arthroplasty" OR "total hip arthroplasty" OR "hemiarthroplasty" OR "THA" OR "HA" OR "total hip replacement" OR "hip arthroplasty")

**CENTRAL**

(proximal femoral nail) OR (proximal femoral nail antirotation) OR (intramedullary nail) OR (cephalomedullary nail) OR (PFNA) OR (PFN) in All Text AND (hip arthroplasty) OR (total hip arthroplasty) OR (hemiarthroplasty) OR (THA) OR (HA) OR (total hip replacement) in All Text AND (unstable pertrochanteric fracture) OR (unstable pertrochanteric fracture) OR (unstable hip fracture) OR (31A2) OR (31A3)
